# Genome-scale identification of cellular pathways required for cell surface recognition

**DOI:** 10.1101/gr.231183.117

**Published:** 2018-09

**Authors:** Sumana Sharma, S. Josefin Bartholdson, Amalie C.M. Couch, Kosuke Yusa, Gavin J. Wright

**Affiliations:** 1 Cell Surface Signalling Laboratory, Wellcome Trust Sanger Institute, Cambridge CB10 1SA, United Kingdom;; 2 Stem Cell Genetics Laboratory, Wellcome Trust Sanger Institute, Cambridge CB10 1SA, United Kingdom

## Abstract

Interactions mediated by cell surface receptors initiate important instructive signaling cues but can be difficult to detect in biochemical assays because they are often highly transient and membrane-embedded receptors are difficult to solubilize in their native conformation. Here, we address these biochemical challenges by using a genome-scale, cell-based genetic screening approach using CRISPR gene knockout technology to identify cellular pathways required for specific cell surface recognition events. By using high-affinity monoclonal antibodies and low-affinity ligands, we determined the necessary screening parameters, including the importance of establishing binding contributions from the glycocalyx, that permitted the unequivocal identification of genes encoding directly interacting membrane-embedded receptors with high statistical confidence. Importantly, we show that this genome-wide screening approach additionally identified receptor-specific pathways that are required for functional display of receptors on the cell surface that included chaperones, enzymes that add post-translational modifications, trafficking proteins, and transcription factors. Finally, we demonstrate the utility of the approach by identifying IGF2R (insulin like growth factor 2 receptor) as a binding partner for the R2 subunit of GABA_B_ receptors. We show that this interaction is direct and is critically dependent on mannose-6-phosphate, providing a mechanism for the internalization and regulation of GABA_B_ receptor signaling. We conclude that this single approach can reveal both the molecular nature and the genetic pathways required for functional cell surface display of receptors recognized by antibodies, secreted proteins, and membrane-embedded ligands without the need to make any prior assumptions regarding their biochemical properties.

Membrane-compartmentalized cells receive instructional information from their surroundings by extracellular signaling cues that are often initiated by specific binding events made by plasma membrane–embedded receptors. These extracellular interactions are crucial for the normal development and function of multicellular organisms and can be exploited therapeutically because they are directly accessible to soluble drugs such as monoclonal antibodies (mAbs) (Weiner 2015). Investigating extracellular cell signaling interactions mediated by membrane receptor proteins can be challenging because the proteins are amphipathic, making them difficult to solubilize in their native conformation and because the interactions are typified by weak interaction affinities; consequently, most commonly used methods are generally unsuitable to detect this class of protein interactions (Wright 2009). The biochemical features of low-affinity membrane receptor interactions have necessitated the development of bespoke techniques to detect them, and one approach involves expressing the entire ectodomain of a receptor as a soluble recombinant protein. The ectodomains are usually purposefully oligomerized so that they can be used as highly avid probes to identify binding partners by expression cloning or biochemical purifications (Wright et al. 2010). More recently, we and others have developed large-scale systematic methods to identify novel receptor–ligand interactions by screening for direct interactions within large protein libraries containing hundreds of receptor ectodomains using ELISA (enzyme-linked immunosorbant assay)-style approaches (Bushell et al. 2008; Ozkan et al. 2013; Visser et al. 2015). While successful, this general approach has drawbacks that prevent its wider use by most laboratories because compiling protein libraries containing hundreds of proteins is resource intensive, and most researchers’ interests are usually focused on a single or small number of proteins rather than the networks of interactions within receptor protein families. Importantly, this technique requires that the receptor binding function is retained when expressed by heterologous cells out of the context of the plasma membrane as a soluble recombinant protein. While this is generally the case for proteins that span the membrane once, this is more difficult for receptor complexes and membrane proteins that span the membrane multiple times, presenting additional challenges to characterize their interactions. Moreover, methods detecting binding events between recombinant proteins do not account for the complex environment in which receptor interactions would normally occur at the cell surface, which includes contributions from a charged glycocalyx of carbohydrates and lipids displayed on a dynamic membrane.

The recent development of cell-based genetic screening approaches using highly efficient CRISPR methods now presents the possibility to interrogate the genetic basis of cellular phenotypes on a genome-wide scale (Koike-Yusa et al. 2014; Shalem et al. 2014, 2015; Wang et al. 2014). Libraries of cells that contain biallelic targeted loss-of-function alleles can be created, and by selecting those cells with a phenotype of interest, the gene products involved can be identified (Ma et al. 2015; Parnas et al. 2015; Zhang et al. 2016). Here, we use genome-scale, cell-based CRISPR knockout (KO) screens to determine the molecular basis of cell surface recognition events made by mAbs, secreted proteins, and receptors. We show that this technique is able to not only identify genes encoding cell surface proteins that directly interact with these binding probes but also reveal receptor-specific pathways required for receptor display at the cell surface in a functional form, including enzymes required for essential post-translational modifications, chaperones, and trafficking proteins.

## Results

### Genetic determinants of mAb cell surface epitope display by genome-scale CRISPR screens

**Figure 1.**
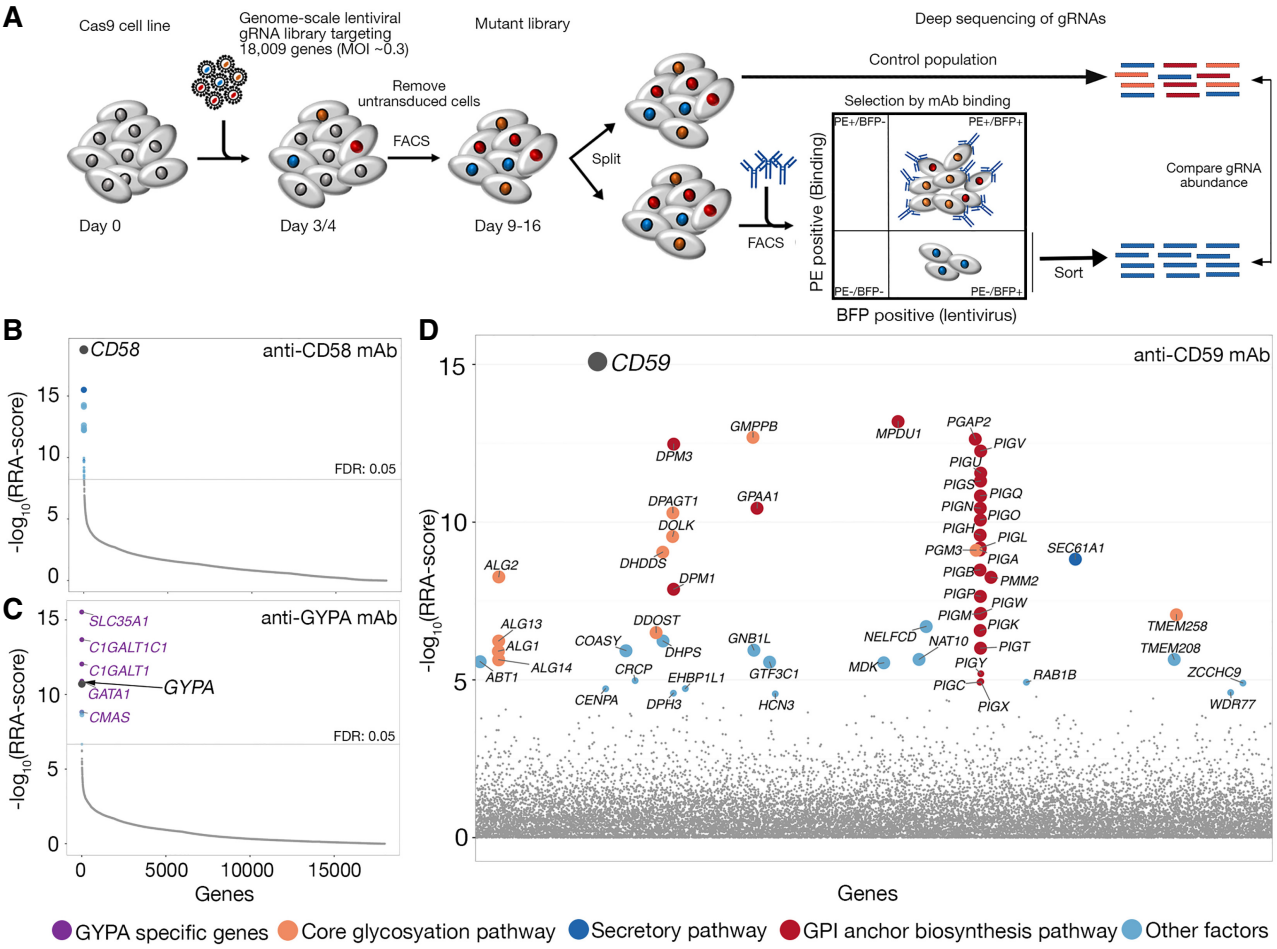
A cell-based genome-scale CRISPR-KO approach identifies pathways required for monoclonal antibody surface epitope recognition. (*A*) Schematic of the approach based on CRISPR/Cas9 technology using a genome-scale lentiviral gRNA library. Genes identified as being required for surface display of the epitope recognized by an anti-CD58 (*B*) and anti-GYPA (*C*) mAbs. The enrichment of gRNAs targeting each gene is quantified as the robust rank aggregation (RRA) score calculated using the MAGeCK software between selected cells that had lost the mAb epitope versus control cells and is shown plotted in rank order. (*D*) Genes identified as being required for surface display of the epitope recognized by an anti-CD59 mAb; here, genes are ordered alphabetically for clarity. Circles represent individual genes and are sized according to their false-discovery rate (FDR): large circle = FDR < 1%, small circle = 1% < FDR < 5%. Genes encoding the direct receptors are indicated with gray circles. Only genes with FDR < 5% are named and are color-coded according to their function.

To determine if a genome-scale, cell-based CRISPR-KO approach could identify genes that are required for specific cell surface recognition events within the context of a plasma membrane, we first selected a panel of six mAbs that brightly stained five different cell surface receptors (Supplemental Table S1). To create a population of mutant cells, the Cas9-expressing cells were transduced at a low multiplicity of infection (∼0.3) with a library of lentiviruses, each encoding a single gRNA from a pool of 90,709 individual gRNAs targeting 18,009 human genes (Tzelepis et al. 2016). Transduced cells that had lost the antibody epitope at the cell surface were isolated by FACS, and the genes responsible for this loss of binding were identified by comparing the relative abundance of the different gene-specific gRNAs present in the sorted cells compared with the total unsorted population using deep sequencing of gRNA PCR products and enrichment analysis (Fig. 1A; Li et al. 2014). Initial experiments established that the day of selection, the number of sorted cells, and sorting thresholds influenced the success of the approach (see Methods), as well as selecting high-activity clonal Cas9-expressing versions for each cell line to homogenize and maximize the efficiency of genome editing (Supplemental Fig. S1). By using this optimized approach, gRNAs targeting the gene encoding the antibody receptor epitope were specifically enriched in the sorted cells for each of the six antibodies (false-discovery rate [FDR] <0.05) (see Supplemental Table S1; Supplemental Data S1). In the case of selections using anti-CD58, gRNAs targeting *CD58* were the most highly enriched (Fig. 1B). In cells selected with the anti-glycophorin A mAb, the most enriched genes targeted *GYPA* encoding the receptor, as well as genes required for sialylated O–linked glycan biosynthesis (*C1GALT1*, *C1GALT1C1*, *SLC35A1*, *CMAS*)—which are presumably critical for creating the extracellular antibody epitope—and the erythroid-specific transcription factor GATA1, which is likely to be necessary for *GYPA* transcription in these cells (Fig. 1C). Similarly, a mAb recognizing the glycosylphosphatidylinositol (GPI)-anchored CD59 receptor identified not only *CD59* as the gene with the most enriched gRNAs but also 21 out of 27 enzymes known to be required for GPI-anchor biosynthesis (Fig. 1D). In addition, a mAb recognizing the α2β1 integrin identified *ITGB1* and components of the Arp2/3 complex, demonstrating the epitope of this mAb is located within the β1 and not α2 chain, and the critical requirement of actin regulation, consistent with the known cell biology of integrin function (Supplemental Table S1; Brakebusch and Fassler 2003). A pathway analysis of all enriched genes that were shared between antibody selection encoded proteins required for protein secretion and glycosylation, as expected, but also identified housekeeping pathways such as ribosome biosynthesis and RNA metabolism (Supplemental Fig. S2). We observed that the representation of these pathways was often reduced when selections were performed several days later, suggesting these genes are required for long-term cell viability in culture and that antibody staining was reduced on moribund cells. An independent repeat of the selections using the anti-CD59 antibody again identified *CD59* as one of the most enriched genes together with genes involved in the GPI-anchor biosynthesis pathway, showing that the experimental parameters were tolerant of biological variation (Supplemental Fig. S3; Supplemental Data S1). Together, these data demonstrated that the genome-scale CRISPR-KO screening approach could not only robustly identify the gene encoding the antibody epitope for all of the six antibodies tested but also reveal genes and pathways that are important in the cell biology for specific receptors.

### CRISPR-KO screening identifies receptors and a role for heparan sulfate in low-affinity interactions

**Figure 2.**
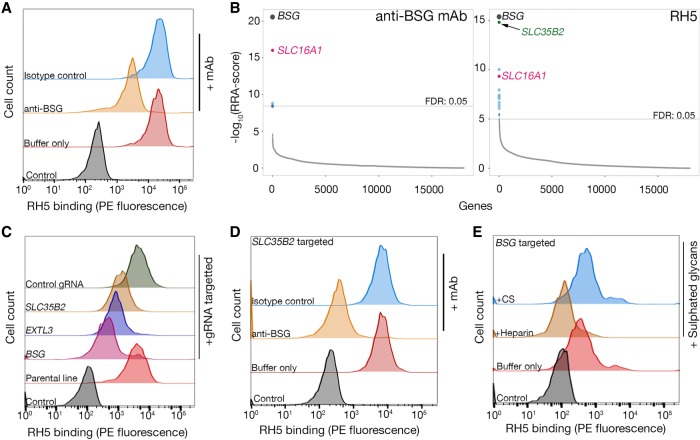
Identification of BSG and heparan sulfate as independent receptors for *P. falciparum* RH5 on HEK293 cells. (*A*) Biotinylated RH5 was clustered around a streptavidin–PE conjugate and binding to HEK293 cells was analyzed by flow cytometry. RH5 binding is only partially reduced by a blocking anti-BSG mAb relative to controls. (*B*) Rank-ordered genes identified from gRNA enrichment analysis required for cell surface display of an anti-BSG mAb (*left*) and RH5 binding (*right*). Significantly enriched genes with a FDR < 0.05 are colored (full screening results available in Supplemental Data S2); genes encoding the receptor (*BSG*) and chaperone (*SLC16A1*) were common to both screens, and a gene involved in GAG-biosynthesis (*SLC35B2*) was additionally required for RH5 binding. (*C*) Binding of RH5 to cells is reduced when transduced with lentiviruses encoding gRNAs targeting either the receptor (*BSG*) or enzymes required for HS synthesis (*SLC35B2*, *EXTL3*) relative to controls. Transduced polyclonal lines were used for this experiment. (*D*) RH5 binding to *SLC35B2*-targeted HEK293 cells could be completely prevented if preincubated with a blocking anti-BSG mAb but not an isotype-matched control. (*E*) RH5 binding to *BSG*-targeted HEK293 cells could be completely blocked if preincubated with 200 µg/mL heparin but not 200 µg/mL CS. A representative of three independent (*A* and *C*) or technical (*D* and *E*) replicate experiments is shown.

While high-affinity mAbs are useful research tools and a few studies have previously shown the utility of CRISPR screens to identify receptor-related cellular pathways (Parnas et al. 2015; Zotova et al. 2016; Burr et al. 2017), we next sought to determine if this approach could be used to identify low-affinity receptors for cell signaling ligands. As a model system, we selected the interaction between *Plasmodium falciparum* RH5 and its host receptor basigin (BSG) because it is a low-affinity interaction (*K*_D_ ∼ 1 µM), is biochemically and structurally well characterized and because BSG was highly expressed on our Cas9-expressing HEK293 cell line (Crosnier et al. 2011; Wright et al. 2014). To detect low-affinity interactions, we increased binding avidity by clustering biotinylated RH5 around a fluorescent streptavidin conjugate, and this reagent bound to the surface of HEK293 cells as expected; however, precoating the cells with a blocking anti-BSG mAb did not prevent all RH5 binding, suggesting there was an additional receptor(s) for RH5 on HEK293 cells (Fig. 2A). This additional binding was not due to a subfraction of inactive protein in the RH5 preparation since all binding could be prevented by heat treatment (Supplemental Fig. S4A). To identify the receptor(s) for RH5 other than BSG in this cellular context, we compared the genes required for RH5 binding versus those necessary for surface expression of BSG by using an anti-BSG mAb. The enriched gRNAs common to both selections beyond those targeting general secretory pathway genes corresponded to *BSG*, as expected, but also to a gene encoding a monocarboxylate transporter, SLC16A1, which is a known chaperone required for surface expression of BSG (Fig. 2B; Kirk et al. 2000). The most highly enriched gene in the cells sorted using RH5 compared with anti-BSG was *SLC35B2* (solute carrier family 35 member B2), which encodes a protein that transports 3′-phosphoadenosine-5′-phosphosulfate from the cytosol into the lumen of the Golgi apparatus, where sulfotransferases use it as a universal donor for the sulfation of major constituents of the cellular glycocalyx, including glycoproteins, glycolipids, and glycosaminoglycans (GAGs) (Fig. 2B; Kamiyama et al. 2003). An analysis of the enriched genes required for RH5 binding using KEGG (Kanehisa et al. 2017) identified the heparan sulfate (HS) biosynthesis pathway, and consistent with this, RH5 binding could be inhibited to a threshold value by heparin, but not the related GAG, chondroitin sulfate (CS) (Supplemental Fig. S4B,C). This is in agreement with the reported presence of heparin binding motifs in RH5 and its ability to bind heparin-coated agarose (Baum et al. 2009). HS is a component of the cellular glycocalyx surrounding most cells and is known to adsorb a wide range of extracellular proteins both as a coreceptor for signaling proteins and to interact with the extracellular matrix (Esko et al. 2009). To further investigate the role of SLC35B2 and HS in RH5 binding, we first demonstrated that surface expression of BSG was not affected in cells where genes required for GAG biosynthesis were targeted (Supplemental Fig. S4D). We then showed that cells targeted for genes required for HS biosynthesis (*SLC35B2* and *EXTL3*) showed a partial reduction in RH5 binding (Fig. 2C) and that the residual binding was specifically due to BSG because it could be completely abrogated by preincubating the cells with the blocking anti-BSG antibody (Fig. 2D). This suggested that the contributions of BSG and HS to RH5 binding were independent, and this was further confirmed by showing that soluble heparin, but not CS, could block all RH5 binding to BSG-deficient cells (Fig. 2E). These experiments revealed a role for HS within the glycocalyx for interactions at the cell surface and demonstrated that this technique was able to identify the direct receptor of low-affinity ligands, including any required receptor cofactors such as chaperones.

### Genome-scale, cell-based CRISPR-KO screens identify directly interacting receptors

**Figure 3.**
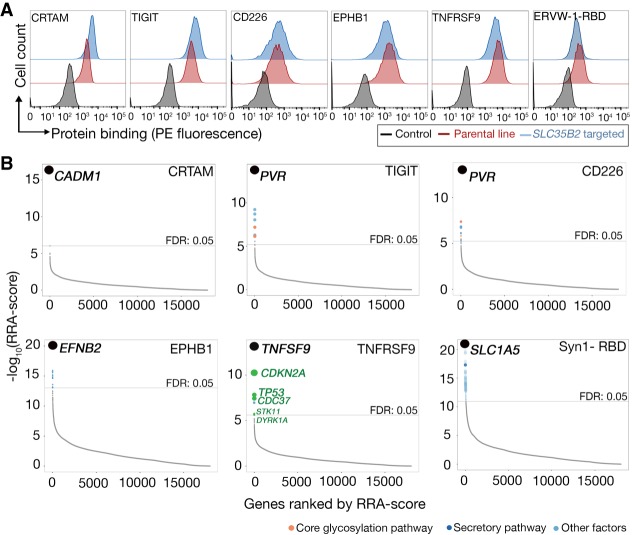
Identification of cell adhesion receptors and pathways using cell-based genetic screens. (*A*) The indicated oligomeric probes were tested for binding to the unmodified parental cell line (red histograms) or polyclonal *SLC35B2*-targeted cells (blue); representative experiments of three technical replicates are shown. The HEK293 cell line was used for all proteins except TNFRSF9, which is the NCI-SNU-1 line. (*B*) RRA-score rank-ordered genes identified from gRNA enrichment analysis from sorted cells that had lost binding to CRTAM, TIGIT, CD226, EPHB1, TNFRSF9, and ERVW-1 (ERVW-1-RBD); in all six cases, the gene encoding the known receptor was identified as the most significantly enriched gene. In *B*, genes with FDR < 5% are labeled with colors corresponding to related functions (full screening results available in Supplemental Data S3).

Many extracellular proteins are known to bind HS, and the finding that HS binding in our assay could be additive rather than codependent on other receptors suggested that HS may represent a factor responsible for cell surface binding for a range of ligands even in the absence of another receptor. To examine and address this, we took advantage of our *SLC35B2*-targeted cell line to rapidly determine the contribution of cell staining due to extracellular sulfate adsorption by comparing ligand binding events between the parental and *SLC35B2*-targeted cells. By using this approach, we identified six ligands with known protein receptors, which, when presented as avid binding reagents, bound HEK293 cells and exhibited no loss of staining when *SLC35B2* had been targeted. These ligands were as follows: CRTAM, TIGIT, CD226, EPHB1, TNFRSF9, and N-terminal receptor binding domain (RBD) of ERVW-1 (also known as Syncytin 1) (Fig. 3A). When these protein probes were used in our CRISPR screening approach, the gene with the most enriched gRNAs corresponded to a known receptor in each and every case: *CADM1* was the top-ranked gene when selected with CRTAM, *PVR* for both TIGIT and CD226 ligands, *EFNB2* for EPHB1, *TNFSF9* for TNFRSF9, and *SLC1A5* for the RBD of ERVW-1 (ERVW-1-RBD) (Fig. 3B). For TNFRSF9, several genes involved in the p53 pathway (*CDKN2A*, *CDC37*, *STK11*, and *DYRK1A*) and *TP53* itself were also enriched in the nonbinding population, suggesting a role for the p53 pathway in presenting TNFSF9 in a functional ligand-binding form on the cell surface. We validated this by independently targeting *TP53*, which resulted in a decrease in the binding of the TNFRSF9 ligand (Supplemental Fig. S5A). An independent repeat of the selections using the TIGIT protein again identified the gene encoding its receptor, PVR, as the most highly enriched gene, demonstrating that the experimental parameters were tolerant of biological variation (Supplemental Fig. S5B). These experiments demonstrate that the directly interacting receptor could be unambiguously identified with high statistical confidence in every case, and for some proteins, the cellular pathways responsible for the cell biology of the receptor can be determined.

### IGF2R interacts with GABBR2 in a M6P-dependent manner

**Figure 4.**
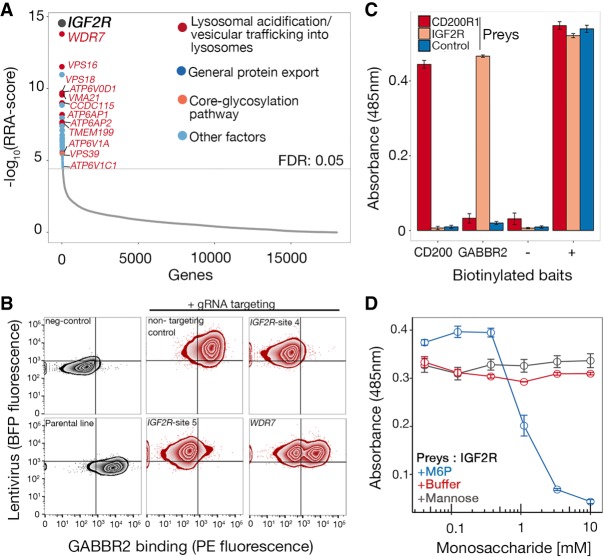
IGF2R interacts with GABBR2 in a mannose-6-phosphate (M6P)–dependent manner. (*A*) RRA-score rank-ordered genes identified from gRNA enrichment analysis from sorted mutant cells that had lost GABBR2 binding activity. Enriched genes encoded the IGF2R receptor and proteins involved in lysosome biology (also see Supplemental Data S3). (*B*) Binding of GABBR2 was quantified on HEK293 cells transduced with two gRNAs targeting different exons of *IGF2R* and one gRNA targeting *WDR7*. A near complete loss of binding was observed on *IGF2R*-targeted cells and a partial loss on *WDR7*-targeted cells; targeted cells were maintained as polyclonal lines. A representative experiment of three technical replicates is shown. (*C*) Direct binding between IGF2R and GABBR2 ectodomains. The biotinylated ectodomain of GABBR2 was immobilized as a “bait” on streptavidin-coated microtiter plates and tested for direct interactions using a beta-lactamase–tagged “prey” ectodomain of IGF2R. Binding was quantified using the beta-lactamase substrate nitrocefin, whose hydrolysis products absorb at 485 nm. Positive control was the CD200–CD200R1 interaction; control “prey” is an unrelated ectodomain, positive (+) represents total capture of all preys with an anti-prey antibody, and negative (−) represents a tag-only bait control. (*D*) The interaction between IGF2R and GABBR2 can be completely inhibited by 10 mM soluble M6P. The binding dependency on M6P was tested by adding serial dilutions of mannose, M6P, or buffer alone. Data points in *C* and *D* are mean ± SEM, *n* = 3.

The results of these experiments suggested that the cell-based, genome-scale CRISPR-KO approach could be used to identify not only directly interacting cell surface receptors but also the cellular pathways required for functional receptor presentation at the cell surface. To demonstrate this, we identified the gamma-aminobutyric acid (GABA) type B receptor subunit 2 (GABBR2) as a receptor whose ectodomain bound HEK293 cells in an *SLC35B2*-independent manner. GABBR2 has an important but mechanistically poorly understood role in the internalization of inhibitory GABA_B_ receptor complexes to regulate neurotransmission (Hannan et al. 2011; Benke 2013). Sorted mutant cells that had lost the ability to bind the GABBR2 ectodomain were enriched in gRNAs targeting the gene *IGF2R* (insulin like growth factor 2 receptor, also known as the cation-independent mannose-6-phosphate (M6P) receptor and CD222), as well as several genes encoding proteins required for endosomal trafficking and function (Fig. 4A). These genes included subunits of V-type ATPases (*ATP6V0D1*, *ATP6AP1*, *ATP6AP2*, *ATP6V1A*, *ATP6V1C1*), a chaperone required for V-ATPase assembly (*VMA21*), V-ATPase assembly factors (*TMEM199*, *CCDC115*), an accessory factor involved in endosomal acidification (*WDR7*), and proteins required for membrane trafficking (*VPS16*, *VPS18*) and fusion (*VPS39*). IGF2R is a known cargo receptor that transports M6P-modified proteins between the *trans*-Golgi network, endosomes, and plasma membrane and might therefore provide a mechanism for the known internalization and degradation of GABA_B_ receptors through interactions with the GABBR2 subunit (Grampp et al. 2007). To investigate this further, we first showed using immunocytochemistry that both proteins were localized within the membranous compartments of cells with IGF2R localizing as discrete puncta in internal membranes that also contained GABA_B_ receptors (Supplemental Fig. S6A). To further investigate the interaction between these two proteins, we first validated our results using individual gRNAs targeting *IGF2R*, which resulted in the loss of both IGF2R surface staining (Supplemental Fig. S6B) and binding of the GABBR2 ectodomain (Fig. 4B). It is known that cells treated with compounds that increase lysosomal pH cause IGF2R to accumulate in endosomes with a consequent loss from the cell surface, providing an explanation for why genes required for endosomal function and trafficking were also enriched (Reaves and Banting 1994). Consistent with this, individual gRNAs targeting the *WDR7* gene, which is required for lysosomal acidification (Merkulova et al. 2015), resulted in the reduction of both IGF2R mAb staining (Supplemental Fig. S6B) and GABBR2 ectodomain binding (Fig. 4B). To demonstrate that the loss of GABBR2 binding to cells was due to the direct interaction with IGF2R, we first showed that GABBR2 ectodomain binding could be conferred to cells by transfecting them with an expression plasmid encoding an IGF2R-GFP fusion protein (Supplemental Fig. S6C) and then observed that the entire ectodomains of IGF2R expressed as a soluble beta-lactamase–tagged “prey” could be specifically captured by a biotinylated GABBR2 ectodomain “bait” (Fig. 4C). We further demonstrated that the interaction between IGF2R and GABBR2 was dependent on M6P-modified N-linked glycans by showing the interaction was abolished by either treating the GABBR2 ectodomain with PNGase F (Supplemental Fig. S6D) or adding soluble M6P (Fig. 4D). These results demonstrate that the ectodomain of GABBR2 interacts directly with IGF2R in a M6P-dependent manner.

### IGF2R is a trafficking receptor for GABBR2

**Figure 5.**
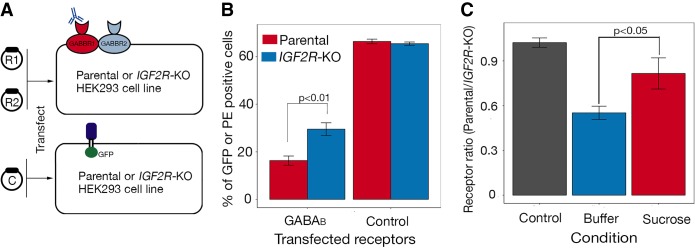
IGF2R traffics GABBR2 through a clathrin-dependent pathway. (*A*) Schematic of a flow cytometry–based GABA_B_ cell surface expression assay. Parental and *IGF2R*-KO HEK293 cells were cotransfected with plasmids encoding both *GABBR1* (R1) and *GABBR2* (R2) full-length cDNAs and surface expression of GABA_B_ receptors quantified by antibody staining and FACS. A plasmid containing a GFP-tagged *CD200* cDNA was transfected in parallel and the number of transfected cells quantified by GFP fluorescence. (*B*) The percentage of GABA_B_ receptor–positive cells was significantly higher on the surface of *IGF2R*-KO cells compared with the parental line (*P* < 0.01 using Welch's two sample *t*-test, *n* = 3); control demonstrates both cell lines were transfected at comparable levels. Error bars, ± SD, *n* = 3 (cells were transfected in triplicate); a representative experiment of two independent experiments is shown. (*C*) The clathrin-pathway inhibitor sucrose was added to parental and IGF2R-deficient HEK293 cells expressing the GABA_B_ receptor complex, and the cell surface receptor levels were quantified. The effect of sucrose on the IGF2R–GABA_B_ receptor complex interaction was determined by calculating the ratio of positively stained cells on the parental cell line relative to the IGF2R-deficient cells and a buffer-only control. Sucrose treatment led to a significant increase (*P* < 0.05 using Welch's two sample *t*-test) in cell surface GABA_B_ receptor complex staining on the parental cell line, almost to the same level as on the IGF2R-deficient cells, demonstrating that that GABA_B_ receptor trafficking depends on the clathrin pathway. The control represents a transfection control to demonstrate parental and IGF2R-deficient cells were transfected with equal efficiency. Bars, mean ± SD; *n* = 3 (cells were transfected in triplicate).

Based on what is already known about the GABA_B_ receptor complex and IGF2R, we hypothesized that this interaction provided a possible molecular mechanism to explain how GABA_B_ receptors are internalized from the cell surface to regulate their activity. To test this, we first targeted *IGF2R* in Cas9-expressing HEK293 cells and selected an IGF2R-deficient clonal line (Supplemental Fig. S7A). To determine how the cell biology of the GABA_B_ receptor complex was altered in the absence of IGF2R, we transfected the cells with cDNAs encoding both subunits of the GABA_B_ receptor and quantified the amount of GABA_B_ receptor complex on the surface of unpermeabilized cells (Fig. 5A). We observed that the average amount of cell surface GABA_B_ receptor complex was significantly higher in the *IGF2R*-KO cell line (Fig. 5B; Supplemental Fig. S7B,C), which is consistent with the model that GABA_B_ receptor complex internalization is promoted by IGF2R. It has also been previously shown that GABA_B_ receptor internalization depends on clathrin-mediated internalization as treatment of cells with hypertonic concentrations of sucrose or chlorpromazine, which have been shown to inhibit the formation of clathrin-coated pits, leads to a complete block of internalization of GABA_B_ receptors from the surface of HEK293 cells (Grampp et al. 2007). To assess the influence of the clathrin-dependent pathway on the internalization of the GABA_B_ receptors, we used our cell-based functional IGF2R–GABA_B_ complex interaction assay. We observed that upon treatment of cells with sucrose, the average amount of cell surface GABA_B_ receptor complex increased on a population of parental HEK293 cells relative to a control demonstrating that GABA_B_ receptor trafficking depends on the clathrin pathway (Fig. 5C). Additionally, to investigate the effect of the agonist GABA on the interaction of the IGF2R–GABA_B_ complex, we added GABA to both the parental and IGF2R-deficient cells expressing the GABA_B_ receptor complex and quantified the IGF2R-dependent internalization of the complex using our cell-based assay. We observed that in the presence of GABA, there was no significant difference in the ratio of cell surface GABA_B_ receptor complex between the parental and IGF2R-deficient cells, demonstrating that GABA agonists have no effect on the IGF2R–GABA_B_ receptor complex interaction (Supplemental Fig. S8). These data demonstrate that the IGF2R-mediated internalization of the GABA_B_ receptor complex is clathrin dependent, and provide evidence that IGF2R is involved in the internalization of GABA_B_ receptors through direct interactions with the GABBR2 receptor subunit.

## Discussion

Here we have shown how it is possible to use a cell-based, genome-scale CRISPR-KO approach to identify genes encoding proteins required for cellular recognition. This genetic approach provides a valuable alternative to existing biochemical methods that must account for the largely insoluble nature of membrane-embedded receptors and the often highly transient nature of their extracellular interactions. This technique has advantages in that it not only is able to reveal the receptor protein that directly interacts with a presented ligand but can also identify other cell-intrinsic gene products that are required for presenting the receptor at the cell surface in an active conformation. In the experiments reported here, this included essential chaperones, actin-interacting proteins, vesicle trafficking adapters, enzymes required for specific glycan biosynthesis, and transcription factors. Genes that were repeatedly identified in different screens had known functional roles in general “house-keeping” receptor biology, including those involved in the secretory and general glycosylation pathways. One important advantage of this approach is that it should be able to identify all gene products required for receptor interactions without the need to make any prior assumptions regarding the cell biology or biochemical nature of the receptor. The genetic nature of the approach may be especially useful to identify interactions involving membrane-embedded proteins that are biochemically very challenging to work with; for example, we show here how this approach can confidently identify the receptor for ERVW-1-RBD, a protein that spans the membrane 12 times and is therefore difficult to solubilize in an active conformation. We anticipate that this single method will be useful to explore the contribution of other molecules such as lipids and may reveal novel pathways for receptor trafficking and biology.

We found that we could additionally use this approach to investigate the contribution to extracellular interactions made by components of the cellular glycocalyx such as GAGs. Many secreted proteins are known to interact with GAGs, especially HS, and similar cell-based genetic screens to identify pathogen receptors have shown that they also play important roles in the interactions of bacteria (Rosmarin et al. 2012) and viruses (Marceau et al. 2016; Pillay et al. 2016) with host cells. The interactions are largely electrostatic with the brush-like negatively charged surface HS forming salt bridges with surface-exposed basic residues and are generally thought to provide a suitable scaffold to present ligands to host receptors in an appropriate manner by regulating their orientation and oligomerization and by establishing local concentration gradients (Yan and Lin 2009). The *P. falciparum* RH5 protein is a typical example of a secreted protein that is known to interact with both heparin-like molecules (Baum et al. 2009) and the BSG receptor (Crosnier et al. 2011), and we were able to clearly identify both in a single experiment using our approach. Further investigation showed that the contributions to RH5 binding by BSG and HS, at least in this context, were independent and could be experimentally separated. This finding enabled us to rapidly establish whether sulfated GAGs played a role in the binding of other ligands by testing binding on an *SLC35B2*-deficient cell line or preincubating the ligand with heparin.

We demonstrated the utility of this technique by identifying IGF2R as a binding partner for GABA_B_ receptors. GABA_B_ receptors are expressed abundantly in almost all types of neurons and glia throughout the central nervous system and mediate slow-acting control of neuron excitability by inhibiting neurotransmitter release. This expression pattern overlaps with that of IGF2R, which is also widely distributed throughout the nervous system with particular enrichment in cortical areas, the hippocampus, and cerebellum (Hawkes and Kar 2004). The regulation of the surface level of GABA_B_ receptors by endocytosis is an important mechanism to attenuate signal strength and can be modelled in HEK293 cells, where GABA_B_ receptors are rapidly and constitutively internalized by the clathrin-dependent pathway (Grampp et al. 2007) to early endosomes. Our finding that IGF2R can directly interact with the GABBR2 subunit of the GABA_B_ receptor complex provides a mechanism for the internalization because IGF2R is itself constitutively endocytosed and trafficked to the endosome compartment through clathrin-mediated uptake via “YSKV” motifs in its cytoplasmic region (Jadot et al. 1992). This is also consistent with the regulatory role for the GABBR2 subunit in the uptake of the GABA_B_ receptor complex (Hannan et al. 2011) and the fact that antibodies directed against the extracellular region, but not receptor agonists, can inhibit GABA_B_ receptor endocytosis (Grampp et al. 2007). A similar role for IGF2R in down-regulating cell surface receptor levels has been shown for CD26 in activated T cells (Ikushima et al. 2000). This finding may now provide new opportunities to regulate GABA_B_ receptor signaling to treat neurological conditions such as epilepsy and depression.

In summary, we have applied a genome-scale, cell-based genetic method based on CRISPR technology to identify genes that mediate cellular recognition processes. We believe that this approach, because it makes no prior assumptions regarding the biochemical nature of the cell surface receptor, will make novel and valuable insights into the molecular basis of cellular adhesion and signaling.

## Methods

### Recombinant protein expression and quantitation

All proteins were expressed in HEK293 cells maintained in FreeStyle media (Life Technologies) supplemented with 50 µg/mL G418 and 0.1% Kolliphor P188 essentially as previously described (Kerr and Wright 2012). For routine culture, 2.5 × 10^7^ HEK293 cells were seeded in 500-mL Erlenmeyer culture flasks containing 100 mL culture media and cultured in a shaker set at 37°C, 5% CO_2_, 70% humidity, and 125 rpm. To maintain a logarithmic growth phase, cells were diluted into fresh media every 2–3 d when the cell density reached ∼2 × 10^6^/mL. HEK293 cells were transiently transfected as previously described (Durocher et al. 2002); briefly, cells were prepared 48 h prior to transfection by seeding at a density of 5 × 10^5^ cells/mL, transfected, and incubated for 5 d before supernatants were harvested by removing cells by centrifugation at 3220*g* for 5 min and cell debris by filtering through a 0.2-µm filter. For the production of biotinylated proteins, the culture media was supplemented with 100 µM D-biotin (Bushell et al. 2008) and cotransfected with a plasmid encoding a secreted *Escherichia coli* BirA enzyme (Addgene plasmid no. 64395) as previously described (Kerr and Wright 2012). Supernatants containing pentameric recombinant proteins were stored at 4°C until further use, whereas the monomeric biotinylated his-tagged proteins were enriched on Ni^2+^-NTA Sepharose beads (Jena Biosciences) using gravity flow chromatography with polypropylene columns with a 50- to 100-µL bed volume (Qiagen). The beads were washed once with wash buffer (20 mM phosphate, 0.5 M NaCl, and 40 mM imidazole at pH 7.4) before the samples were eluted with 300–500 µL of elution buffer (20 mM phosphate, 0.5 M NaCl, and 400 mM imidazole at pH 7.4). Eluted proteins were separated by SDS-PAGE under reducing conditions and visualized with InstantBlue (Expedeon) to confirm their size and integrity following the manufacturer's recommendations. A list of the expression plasmids for human proteins used in this study can be found in Supplemental Table S2.

Biotinylated protein expression levels were quantified by ELISA as previously described (Kerr and Wright 2012). Briefly, proteins were captured on 96-well streptavidin-coated plates (NUNC) for 1 h before adding 10 µg/mL primary antibody recognizing the rat Cd4d3+4 tag (mouse anti-rat Cd4d3+4, clone OX68, Bio-Rad [MCA1022R]) common to all recombinant proteins for another hour. Plates were washed 3× in PBS/0.1% Tween-20 (PBST) before adding 100 µL of an anti-mouse alkaline phosphatase conjugate (Sigma, A3562) at 0.2 µg/mL. Plates were washed 3× PBST and 1× PBS before adding 100 µL *p*-nitrophenyl phosphate (Sigma 104 alkaline phosphatase substrate) at 1 mg/mL in diethanolamine buffer (10% diethanolamine [v/v], 0.5 mM MgCl_2_, 10 mM NaN_3_ at pH 9.8). Absorbance readings were taken 15–20 min post substrate addition at 405 nm on a PHERAstar plus (BMG Laboratories). Beta-lactamase–tagged proteins were quantified using nitrocefin hydrolysis as previously described (Bushell et al. 2008). The proteins were normalized to enzyme activity corresponding to ∼1 nmol/min, which corresponds to complete hydrolysis of 14.5 nmol nitrocefin in ∼15 min.

### Plate-based direct protein interaction assay

A biotinylated “bait” protein consisting of the entire ectodomain of GABBR2 and controls were first immobilized in a well of a streptavidin-coated 96-well microtiter plate (NUNC) at a concentration that saturated the biotin binding capacity of the well and probed for direct interactions with the entire ectodomain of IGF2R expressed as a beta-lactamase–tagged “prey.” The plate was washed 2× in PBST, after which normalized beta-lactamase–tagged “prey” (IGF2R and controls) proteins were added to the wells for 1 h. Following another wash step (2× with PBST and a final wash with only PBS), 100 µL of 125 µg/mL nitrocefin was added, and prey capture was quantified by measuring the absorbance of nitrocefin hydrolysis products at 485 nm on a PHERAstar plus (BMG Laboratories). Biotinylated Cd4d3+4 tag alone was used as a negative control bait, and a biotinylated anti-Cd4 mAb (anti-prey) used as a positive control as required.

Where soluble monosaccharides were used in blocking experiments, prey proteins were first incubated with a range of concentrations (10–0.04 mM) of M6P or mannose for 1 h, prior to incubation with bait proteins. To remove N-linked glycans from soluble recombinant GABBR2, 1500 U of PNGase F (New England Biolabs) was added to 10 µg of GABBR2 and incubated for a duration ranging from 1–16 h at 37°C.

### Cell culture of human cell lines

NCI-SNU-1, HEL, and SK-N-SH cells were cultured in RPMI 1640 (Life Technologies) supplemented with 10% heat-inactivated (50°C for 20 min) FBS, 1 mM sodium pyruvate (Life Technologies), 10 mM D-glucose (Sigma), and penicillin–streptomycin at 37°C with 5% CO_2_. HEK293 cells were cultured in FreeStyle media (Life Technologies) supplemented with 1% heat-inactivated FBS in shaker flasks in a shaking incubator set at 125 rpm, 37°C, 70% humidity, and 5% CO_2_. To maintain a logarithmic growth phase, NCI-SNU-1, HEL, and HEK-293 cells were diluted into fresh media every 2–3 d once the cell density reached ∼1 × 10^6^/mL. SK-N-SH cells were grown in a monolayer, and the culture medium was changed every 3 d. The cells were passaged once they reached ∼80% confluence. NCI-SNU-1 and SK-N-SH cells were obtained from the Sanger Institute Cancer Cell Line Panel (https://cancer.sanger.ac.uk/cell_lines). HEK293 cells were obtained from Dr. Yves Durocher (Durocher et al. 2002). HEL cells were purchased from Deutsche Sammlung von Mikroorganismen und Zellkulturen (DSMZ). All cell lines were tested and found to be mycoplasma free.

### Cell binding assay with recombinant proteins and mAbs

#### Binding assay using biotinylated monomeric proteins

To increase binding avidity, biotinylated monomeric Cd4d3+4-tagged proteins were multimerized around streptavidin–phycoerythrin (PE). To ensure all biotin binding sites on the streptavidin were occupied and to minimize the presence of excess monomer, serial dilutions of biotinylated protein samples were titrated against a fixed concentration of streptavidin–PE (100 µL of 0.1 µg/mL) for 20 min at room temperature before transferring to a streptavidin-coated plate and assaying for the capture of any excess biotinylated Cd4d3+4-tagged proteins using the OX68 ELISA. The minimal dilution at which all biotinylated Cd4d3+4-tagged protein was captured was subsequently used to create tetramers. A 10× tetramer staining solution was prepared using 4 µg/mL streptavidin–PE and the appropriate biotinylated protein dilution by incubating for 30 min at room temperature and then diluted to 1× and 100 µL added to 0.5–1 × 10^6^ cells in an U-bottomed 96-well microtiter plates and incubated for 1 h at room temperature. Where the anti-BSG antibody was used in blocking experiments, cells were first incubated with 10 µg/mL antibody (or isotype-matched control) for 1 h prior to incubation with the RH5–streptavidin–PE complex. Cells were washed once with wash buffer (PBS with Ca^2+^/Mg^2+^ [HyClone, Sigma] supplemented with 1% BSA) and analyzed by flow cytometry.

#### Binding assay using pentameric protein supernatants

The 3xFLAG and beta-lactamase–tagged pentameric proteins were quantified directly from supernatants and normalized to ∼1 nmol/min using the beta-lactamase enzyme activity. Next, 100 µL of diluted proteins was added to 0.5–1 × 10^6^ cells in a U-bottomed 96-well microtiter plates for 1 h at room temperature. Following a wash with wash buffer, 100 µL PE-conjugated anti-FLAG antibody (0.5 µg/mL, Abcam ab72469) was added to the samples and incubated for 1 h. The cells were again washed once in wash buffer and analyzed by flow cytometry.

#### Binding assay with mAbs

For antibody staining of cell surface proteins, 50 µL of 1 µg/mL primary antibody was incubated with 1 × 10^6^ cells in 96-well U bottom plates. The cells were washed after 1 h of primary antibody incubation, after which 100 µL of an appropriate secondary antibody, also conjugated to PE, was used at 0.1 µg/mL. For IGF2R staining, anti-IGF2R mAB-clone-2G11 (Abcam, ab2733) was used. For staining of GABA_B_ receptors, anti-GABA B receptor 1 antibody (Abcam, ab55051) was used.

#### Binding assay with transiently transfected cells

Human IGF2R was expressed on the surface of transfected cells using an expression construct in which its cytoplasmic region was replaced by eGFP, as previously described (Sollner and Wright 2009). NCI-SNU-1 cells, which do not have detectable levels of plasma membrane IGF2R expression, were transiently transfected with either IGF2R-TM-eGFP or CD200R1-TM-eGFP as a control and probed for binding interactions either with GABBR2 ectodomain presented as a tetramer around streptavidin–PE or with an anti-IGF2R mAb (ab2733). Human GABA_B_ receptor heterodimer was expressed on the surface of parental and *IGF2R*-KO HEK293 cell lines by transiently cotransfecting both GABBR1 and GABBR2 cDNA constructs using Lipofectamine 2000 (Invitrogen) according to the manufacturer's protocol. CD200-TM-eGFP was transfected into the cells as a transfection control. The surface level of GABA_B_ receptors was analyzed 3 d after transfection by surface-staining using a GABAB receptor 1 antibody (Abcam, ab55051). Where appropriate, the clathrin-pathway inhibitor sucrose was added at 450 µM. All flow cytometry was performed on a Becton-Dickinson LSRFortessa flow cytometer, collecting between 10,000 to 30,000 events; live cells were gated using forward and side scatter. PE was excited at a wavelength of 561 nm and emission detected using a 582/15 band pass filter; BFP was excited at 405 nm and the emission detected using a 450/50 band pass filter; GFP was excited at 488 nm and the emission detected using 530/30 band pass filter. Analysis was performed using FlowJo software (Treestar).

### Immunofluorescence of SK-N-SH cells

SK-N-SH cells grown as a monolayer in coverslips were fixed in 4% formalin. Fixed cells were permeabilized in PBS/Triton X-100 0.1% and blocked in PBS/2% BSA. Cells were stained with primary antibodies: 1:400 anti-IGF2R (rabbit monoclonal, Abcam 124767) and 1:250 anti-GABBR1 antibody (mouse monoclonal, Abcam ab55051) overnight at 4°C. After three washes in the wash buffer (PBS/2% BSA), the cells were incubated with secondary antibodies (goat anti-mouse IgG [H+L] AlexaFluor 488 [Molecular Probes A-11001], donkey anti-rabbit IgG [H+L] AlexaFluor 647 [Abcam 150075]) diluted 1:500 for 1 h at room temperature. The coverslips were washed twice in wash buffer and three times in PBS and mounted on a microscope glass in SlowFade with DAPI (Molecular Probes) before images were captured with Leica SP5/DM6000 confocal microscope.

### Lentiviral vectors

The Human Improved Genome-Wide Knockout CRISPR Library v1 (Addgene no. 67989), lentiviral Cas9 reporter plasmids pKLV2-U6gRNA5(gGFP)-PGKBFP2AGFP-W (Addgene no. 67980) and pKLV2-U6gRNA5(Empty)-PGKBFP2AGFP-W (Addgene no. 67979), lentiviral vector expressing Cas9 fused with the blasticidin-resistant gene at the C terminus pKLV2-EF1a-Cas9Bsd-W (Addgene no. 68343), and lentiviral CRISPR gRNA expression vector pKLV2-U6gRNA5(BbsI)-PGKpuro2ABFP-W (Addgene no. 67974) were used in this study. All gene-specific gRNAs were cloned into BbsI site of the gRNA expression vector as described previously (Tzelepis et al. 2016).

### Preparation of lentivirus and transductions

Lentivirus were prepared by transfecting HEK293-FT cells as previously described (Koike-Yusa et al. 2014). All Cas9-expressing human cell lines were selected following transduction of cells with lentivirus prepared from the pKLV2-EF1a-Cas9Bsd-W plasmid (Tzelepis et al. 2016). Polybrene (8 µg/mL) was added for the transduction of NCI-SNU-1 and HEL cells. Cells were selected using 20 µg/mL blasticidin for the HEK293 and NCI-SNU-1 cell lines and 10 µg/mL for HEL cells 2 d following transduction. We found that polyclonal Cas9-expressing lines contained a variable fraction (up to 30%) that were refractory to gene editing, which affected the sensitivity of our screens. We therefore always established clonal high Cas9 activity cell lines by sorting individual blasticidin-resistant cells into wells of 96-well plates (MoFlo XDP), which were further expanded and tested for Cas9 activity using the GFP-BFP system (Tzelepis et al. 2016). In brief, cells were transduced with lentivirus encoding GFP, BFP, and a gRNA targeting *GFP* (pKLV2-U6gRNA5(gGFP)-PGKBFP2AGFP-W) or the same construct with an “empty” gRNA (pKLV2-U6gRNA5(Empty)-PGKBFP2AGFP-W) as a negative control. High-activity Cas9 stable cell lines were selected by examining the ratio of BFP only to GFP-BFP double-positive cells transduced by the two lentiviruses. These clonal cell lines were expanded and further tested by targeting an endogenous gene encoding the BSG cell surface protein using lentivirus prepared using a plasmid encoding puromycin, BFP, and a gRNA targeting *BSG* [pKLV2-U6gRNA5(BbsI-g*BSG*)-PGKpuro2ABFP-W]. The surface expression of BSG was quantified by flow cytometry using an anti-BSG mAb (MEM-M6/6, Abcam ab119114) 8 d post transduction to validate high Cas9 efficiency.

#### Lentiviral transduction of HEK293 cells with genome-scale gRNA library

The CRISPR-KO library we used in this study contains 90,709 gRNAs targeting 18,009 human genes at a coverage of five gRNAs per gene (Tzelepis et al. 2016). The backbone vector contains the optimized gRNA scaffold, which can maximize genome editing efficiency. A genome-scale “knock-out” library of HEK293-Cas9 cells was produced by transducing 3 × 10^7^ cells such that ∼30% of the total cell population were transduced to increase the chances that each cell just received a single gRNA. The transduced (BFP-positive) cells were harvested 3 d after transduction using a cell sorter (MoFlo XDP), and libraries containing at least 5 × 10^6^ cells were selected. The libraries were cultured in media containing 2 µg/mL puromycin to remove the contaminated nontransduced cells, and at every passage, at least 10× the initial library (starting cell number on day three) for each library was seeded into new culture flasks. Phenotyping screens for cell surface binding events were carried out between 9 and 16 d post transduction.

#### Lentiviral transduction of NCI-SNU-1 and HEL cells with genome-scale gRNA library

A spinoculation protocol was used to transduce HEL and NCI-SNU-1 cell lines. Two milliliters of 5 × 10^6^ cells/mL was aliquoted in 8 × 15-mL Falcon tubes and mixed with lentivirus together with 8 µg/mL polybrene and incubated at room temperature for 30 min followed by centrifugation for 100 min at 800*g* at 32°C. The supernatant was removed, and the cells from each Falcon tube were resuspended in 50 mL culture media. As with HEK293 cells, cells were sorted on day 3 post transduction to generate control and sample libraries and were grown further in media supplemented with 1 µg/mL puromycin.

#### Lentiviral transduction of human cells with individual gRNAs

For the validation of individual target genes, the corresponding gRNAs from the CRISPR library (Supplemental Table S3) were cloned into the BbsI site of pKLV2-U6gRNA5(BbsI)-PGKpuro2ABFP-W, and lentivirus was produced as described before. Cells (1 × 10^6^ cells/well in six-well plate) were transduced with the lentivirus for at least 24 h. Polybrene (8 µg/mL) was added during transduction of HEL and NCI-SNU-1 cell lines. Fresh media containing puromycin (2 µg/mL for HEK293 cells and 1 µg/mL for all other cell lines) was added to the cells after transduction and cultured for a further 8 d before use in binding assays.

### Cell surface phenotyping, selection, and amplification of selected gRNAs

To identify the genes required for cell surface recognition, the binding of the soluble ligand (mAb or recombinant protein) of interest was first quantified by flow cytometry to a small panel of cell lines, and the cell line exhibiting the highest level of binding was typically selected for genome-wide screening. Genome-scale knock-out libraries were phenotyped by cell surface staining using flow cytometry between 9 and 16 d post transduction. The mutant library was divided into two parts: At least 5 × 10^7^ cells from the mutant library were collected as “control” population for later analysis, whereas 5 × 10^7^–15 × 10^7^ cells from the library were stained with appropriate reagent (recombinant protein or antibody) using the binding assay protocol as described above with minor modifications: Cells (5 × 10^6^ cells/mL) were stained in 15-mL Falcon tubes with gentle rotation (6 rpm), the stained cells were then analyzed using an XDP flow sorter, and the BFP^+^/PE^−^ cells were collected. The percentage of the total library population that was collected in each screen varied between 0.2% and 2.3% and are listed in Supplemental Table S4. We observed that stringent sorting thresholds resulted in the identification of few genes that had strong effects on binding loss, and increasing this threshold enabled the identification of additional genes that presumably had weaker effect sizes on the day of screening. We determined that collecting between 300,000 and 500,000 cells at a 0.5%–1% stringency threshold was generally an appropriate parameter that permitted the identification of genes with weaker effect sizes. All genetic screens except those performed using anti-CD59 mAb and TIGIT recombinant protein in this study were carried out once.

#### Amplification of gRNAs, sequencing, and enrichment analysis

Genomic DNA extraction, PCR enrichment, and Illumina sequencing of gRNAs from both control and sorted samples were carried out as described previously (Koike-Yusa et al. 2014), except in sorted samples where the sorted cell number was less than 100,000. In that case, a cell lysate protocol was used to isolate gRNAs prior to PCR enrichment. Cell lysates were prepared from sorted cells by first aliquoting cells in a 96-well PCR plate (10,000 cells/well) and boiling the samples with 25 µL water for 10 min at 95°C. Next, 5 µL of 2 mg/mL freshly diluted Proteinase K was added to each sample and incubated for 1 h at 56°C and then heated for 10 min at 95°C to inactivate the protease. The gRNAs were then amplified using 10-µL cell lysates per PCR reaction.

For all samples, 19-bp single-end sequencing was performed using a custom sequencing primer 5′-TCTTCCGATCTCTTGTGGAAAGGACGAAACACCG-3′. The read count for each gRNA and gene level enrichment analysis was carried out using the MAGeCK statistical package (version 0.5.5) (Li et al. 2014) by comparing the read counts from the sorted population with those from the control population. Pathway analysis was also carried out using MAGeCK software with default settings with KEGG annotated pathways. All further analysis was carried out using R (R Core Team 2017). For all the target receptors identified, the ranks of the individual gRNAs targeting the related gene are listed in Supplemental Table S5.

### Quantifying gRNA representation in mutant cell libraries to ensure maintenance of library complexity

We observed that it was necessary to culture the cells for between 9 and 16 d after transduction to allow sufficient time for gene targeting and consequent loss of the encoded protein. During this time, gRNAs can be lost from the library for biological reasons (e.g., the gRNA targets a gene essential for cell survival) or for technical reasons (such as the need to passage the library because of cell growth that may create population restriction points reducing gRNA library representation). To ensure that gRNA representation is maintained using our cellular KO library preparation protocol, we quantified the gRNA abundance in at least 5 × 10^7^ cells on different days after transduction from two independent NCI-SNU-1-Cas9 libraries and one library of both HEK293-Cas9 and HEL-Cas9 cells. We observed that gRNA abundances were highly correlated between the biological replicates of NCI-SNU-1-Cas9 libraries, and similarly high correlations were observed among the three different cell line libraries at equivalent time points, demonstrating that the procedure we used to make genome-scale mutant libraries was reproducible (Supplemental Fig. S9). The correlation between the different mutant cell line libraries and the original plasmid population was lower, and so the most appropriate control for gRNA enrichment analysis was the cell line on that day rather than the population of gRNAs in the original plasmid population.

To further compare the gRNA abundances, the number of sequence reads for each gRNA between the original plasmid pool and each cell library was quantified. In the plasmid library, while a small fraction of gRNAs was under- or overrepresented, >82% of the gRNAs were uniformly distributed, with only an eightfold difference in abundance between the 10th and 90th percentiles. The cell-based mutant libraries created from this plasmid library also showed a uniform coverage, although a small drop in the overall representation of the gRNA library was observed in all cell lines as expected, and this decrease was more pronounced when comparing days 9–16 post transduction. It is likely that these depleted gRNAs target genes that are essential for cell growth in culture. To establish this, we performed a gene-level negative selection enrichment analysis to identify genes that were depleted in the mutant library compared with the plasmid library in all 3 d in the NCI-SNU-1 and HEK293 cell lines and on day 14 for HEL cells. As a quality control, we initially analyzed genes required for ribosome biosynthesis (annotations from KEGG-Ribosome), which are known to be essential and are often robustly identified in similar negative selection screens (Wang et al. 2014; Hart et al. 2015). Reassuringly, the majority of gRNAs targeting genes required for ribosome biosynthesis were among the most significantly depleted (FDR < 10%) across all 3 d in all three cell lines (Supplemental Fig. S10). Next, we attempted to identify which pathways were defined by the genes targeted by the enriched gRNAs using KEGG annotated pathways (Kanehisa et al. 2017). We observed that the pathways identified were in biological processes described as being essential, including those required for the spliceosome, cell cycle, purine and pyrimidine biosynthesis, and both DNA and RNA polymerases (Supplemental Data S4). These results provided further confidence that the cellular mutant libraries generated using our protocol retained their gRNA complexity and could be used for genome-scale screening.

## Data access

All reads from the genome-wide screening experiments in this study have been submitted to the European Nucleotide Archive (http://www.ebi.ac.uk/ena) under accession number ERP104831.

## Supplementary Material

Supplemental Material
