# Supplementary material for: Genome-scale identification of cellular pathways required for cell surface recognition
Source: Genome Res. 2018 Sep;28(9):1372–82. doi: 10.1101/gr.231183.117 (PMC6120632; doi:10.1101/gr.231183.117)
Supplement: Supplemental Material [file supp_28_9_1372__index.html]

Genome-scale identification of cellular pathways required for cell surface recognition — Supplemental Material 

# Genome-scale identification of cellular pathways required for cell surface recognition

## Supplemental Material

- Supplemental\_DataS1.zip
- Supplemental\_DataS2.zip
- Supplemental\_DataS3.zip
- Supplemental\_DataS4.zip
- Supplemental\_Figures\_Tables\_Legends.docx
