# Supplementary material for: Genome-scale identification of cellular pathways required for cell surface recognition
Source: Genome Res. 2018 Sep;28(9):1372–82. doi: 10.1101/gr.231183.117 (PMC6120632; doi:10.1101/gr.231183.117)
Supplement: Supplemental Material [file supp_gr.231183.117_Supplemental_Figures_Tables_Legends.docx]

**Supplemental information:** **Genome-scale identification of cellular pathways required for cell surface recognition**

**Supplemental Figure S1:** Selecting clonal cell lines with high Cas9 activity for efficient genome-scale genetic screening.

**Supplemental Figure S2:** Genes required for protein export, N-glycan biosynthesis and general housekeeping functions were enriched in, and shared between, cells selected for the loss of cell surface mAb staining.

**Supplemental Figure S3:** The experimental parameters established to identify cell surface receptor interactions using a cell-based CRISPR screening approach are robust and repeatable.

**Supplemental Figure S4:** RH5 binding to HEK293 cells was heat labile and was partially blocked by preincubation with heparin, but not chondroitin sulfate.

**Supplemental Figure S5:** Validation that *TP53* contributes to binding of TNFRSF9 to NCI-SNU-1 cells and repeated screen with a low-affinity ligand demonstrating the robustness of the screening parameters.

**Supplemental Figure S6:** Membrane localization of IGF2R and GABA_B_ receptor complex, validation of gRNAs targeting *IGF2R* and *WDR7*, and biochemical analysis of the IGF2R-GABBR2 interaction.

**Supplemental Figure S7:** Cell-based functional assay in parental and *IGF2R*-KO HEK293 cells demonstrate that IGF2R internalizes GABA_B_ receptor complex in a clathrin dependent manner.

**Supplemental Figure S8:** Addition of the agonist, GABA, has no effect on the interaction between IGF2R and the GABAB receptor complex.

**Supplemental Figure S9:** Genome-scale libraries of mutant cells are reproducibly created using CRISPR-Cas9 technology and lentiviral gRNA delivery.

**Supplemental Figure S10:** gRNAs targeting genes involved in essential biological processes were depleted during culture of the mutant cell library.

**Supplemental Table S1:** False discovery rate (FDR) for identification of the genes encoding the known antibody target in a genome-scale screening approach.

**Supplemental Table S2**: Protein expression plasmids used in this study

**Supplemental Table S3:** Sequences of guide RNAs used in this study

**Supplemental Table S4:** Summary of screening parameters for all screens carried out in this study**.**

**Supplemental Table S5.** Summary of the relative gRNA abundances targeting the identified receptor in the different screens within this study

**Supplemental Data S1:** Summary of gRNA counts and gene level enrichment file from MAGeCK for all mAb screens relating to Supplementary Table 1, Figure 1, and Supplemental Figure S2.

**Supplemental Data S2:** Summary of gRNA counts and gene level enrichment file from MAGeCK for PfRH5 and anti-BSG mAb screen relating to Figure 2.

**Supplemental Data S3:** Summary of gRNA counts and gene level enrichment file from MAGeCK for all recombinant protein screens relating to Figures 3 and 4.

**Supplemental Data S4:** Raw read counts, enrichment analysis and pathway analysis output from MAGeCK using KEGG-annotated pathways for all control samples (2 replicates of NCI-SNU-1 and 1 sample from HEK293 cells and HEL cells each).

In Supplemental Data S1, S2 and S3, raw gRNA counts in control and sorted population is provided in the ‘.raw_counts.txt’ file. Each other file provides gene level enrichment output for positive selection obtained from MAGeCK. Refer to the file title for the identity of the probe used for screening. In Supplementary Data 4, raw counts, gene enrichment analysis from MAGeCK, and pathway enrichment analysis from MAGeCK are separated into each folder. Refer to the file title under each folder for the corresponding data.

**
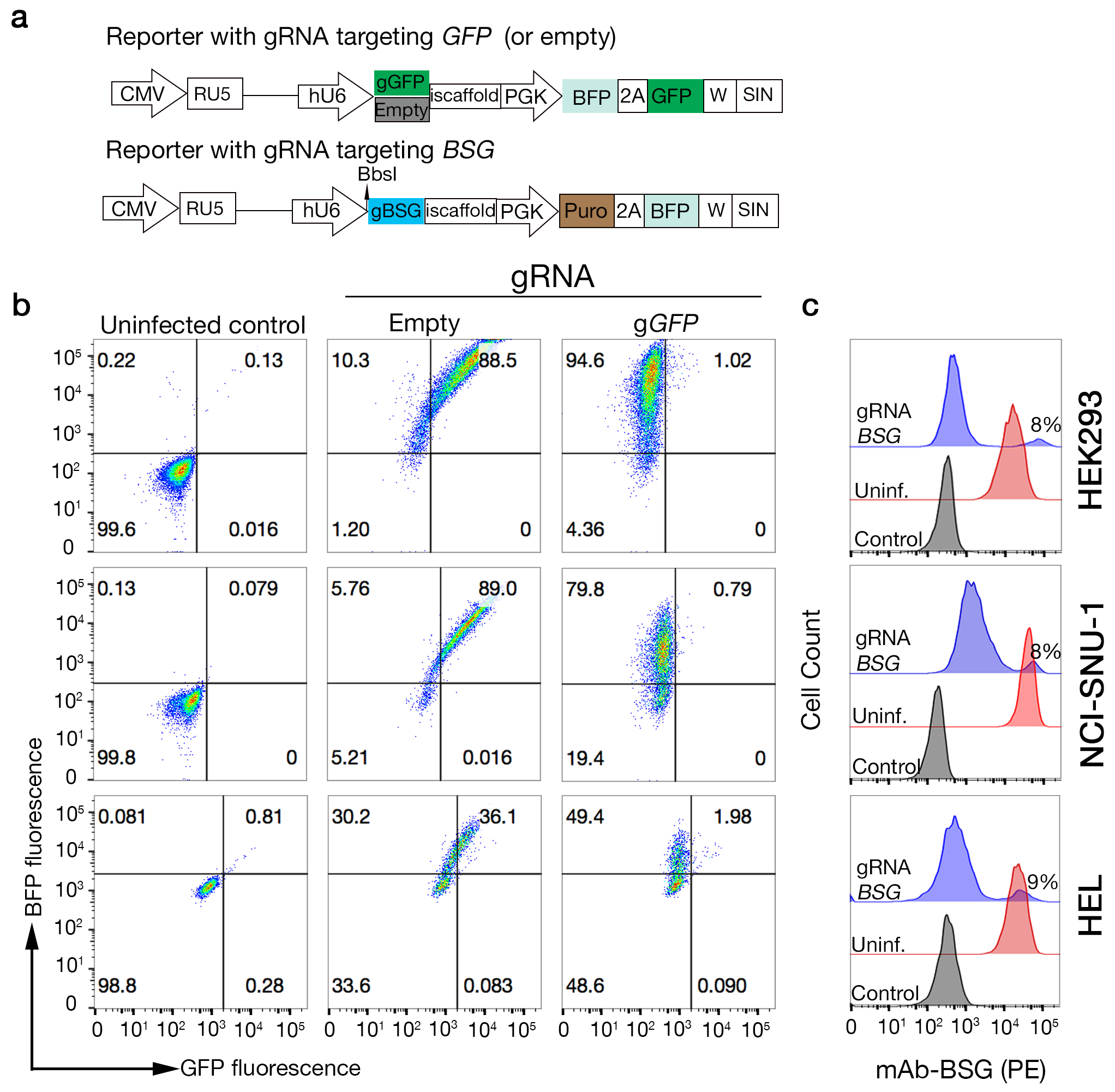
**

**Supplemental Figure S1: Selecting clonal cell lines with high Cas9 activity for efficient genome-scale genetic screening.** Initial experiments using uncloned Cas9-expressing cell lines revealed that a variable fraction of the cell population were refractory to genome editing and so we selected clones that exhibited high Cas9 activity. **a**. Schematics of lentiviral reporter plasmids used to quantify genome editing efficiency in Cas9-expressing cell populations. *GFP*-encoding lentivirus constructs containing either a gRNA targeting *GFP* (gGFP) or not (“empty”) were used to quantify genome editing efficiency in a cloned population of Cas9-expressing cell lines. **b**. Cloned Cas9-expressing HEK293, NCI-SNU-1 and HEL cell lines were tested for both BFP and GFP expression by flow cytometry after infection with lentivirus containing a gRNA targeting *GFP*, no functional gRNA (“empty”), or left uninfected. BFP expression marks transduced cells (note the infection frequency for HEL cells was lower compared to the other two cell lines) and the loss of GFP expression is used to quantify Cas9 activity; the line exhibiting the greatest loss of GFP expression out of at least five clones tested are shown. **c**. The cloned cell lines selected using the GFP-BFP assay were additionally tested for their ability to target an endogenous locus by infecting them with a lentivirus encoding a gRNA targeting *BSG* and quantifying expression of BSG on the cell surface with an anti-BSG mAb; all cell lines exhibited loss of BSG from >90% of the population. Control: parental cell line stained with secondary anti-mouse-IgG-PE alone; Uninf: parental cell line stained with anti-BSG antibody. **b** and **c** are representative experiments of two technical replicates. CMV: Cytomegalovirus promoter, RU5: 5’ long terminal repeat lacking the U3 region, W: Woodchuck Hepatitis Virus posttranscriptional regulatory element, SIN: ∆U3RU5 (self- inactivating 3’ LTR); hU6: human U6 promoter, gGFP: gRNA targeting GFP, gBSG: gRNA targeting BSG, Iscaffold: Improved scaffold, 2A: self-cleaving peptide, PGK: PGK1 promoter, puro: puromycin- resistant gene, BFP: blue fluorescent protein, GFP: green fluorescent protein.

**
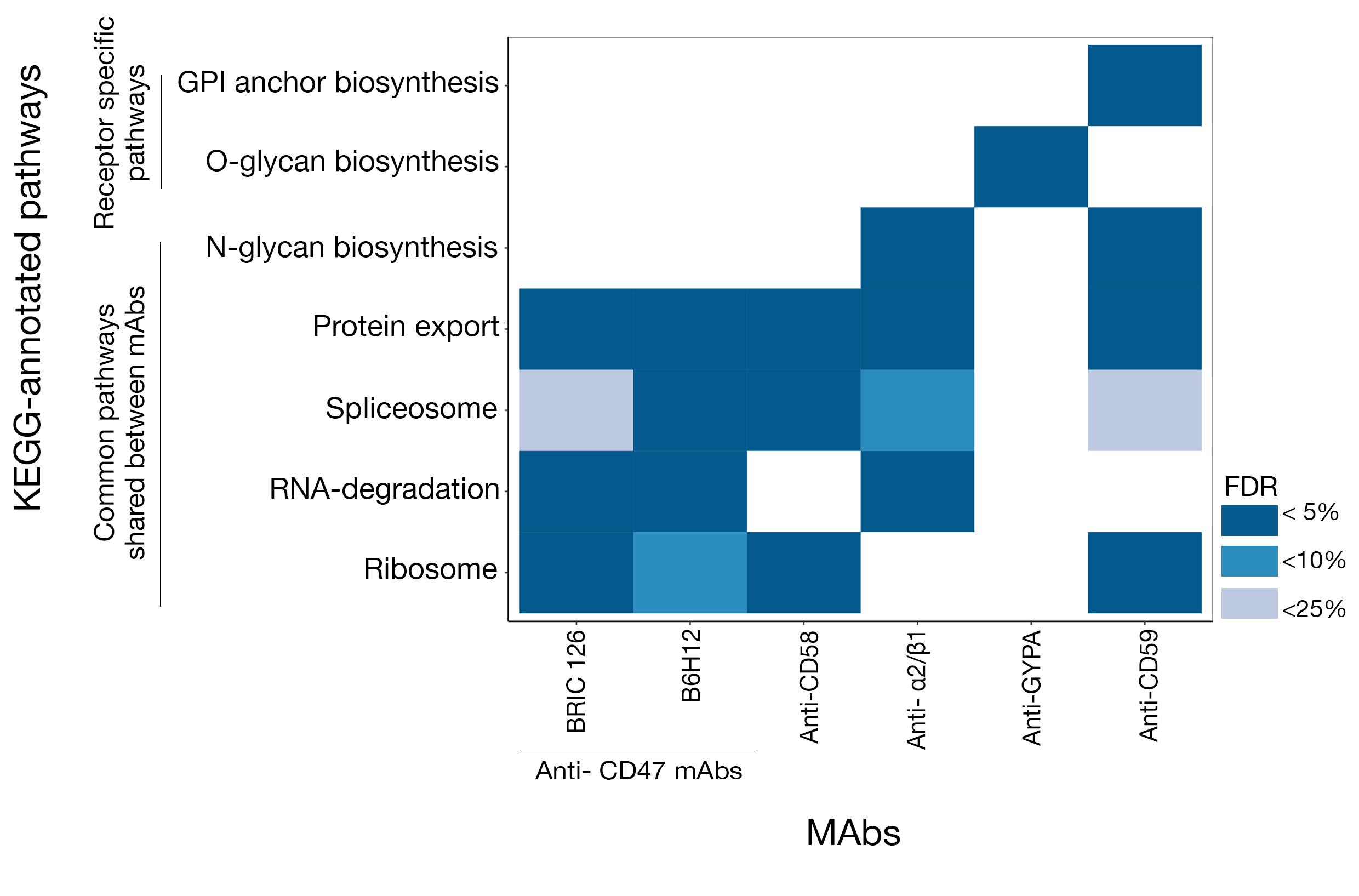
**

**Supplemental Figure S2: Genes required for protein export, N-glycan biosynthesis and general housekeeping functions were enriched in, and shared between, cells selected for the loss of cell surface mAb staining.** Pathway analysis on genes enriched in cells selected with the indicated mAbs identified some pathways shared between more than one antibody including protein export, N-glycan biosynthesis as well as more general housekeeping functions; genes identified in these pathways were grouped and labeled as “other factors” in subsequent analyses.


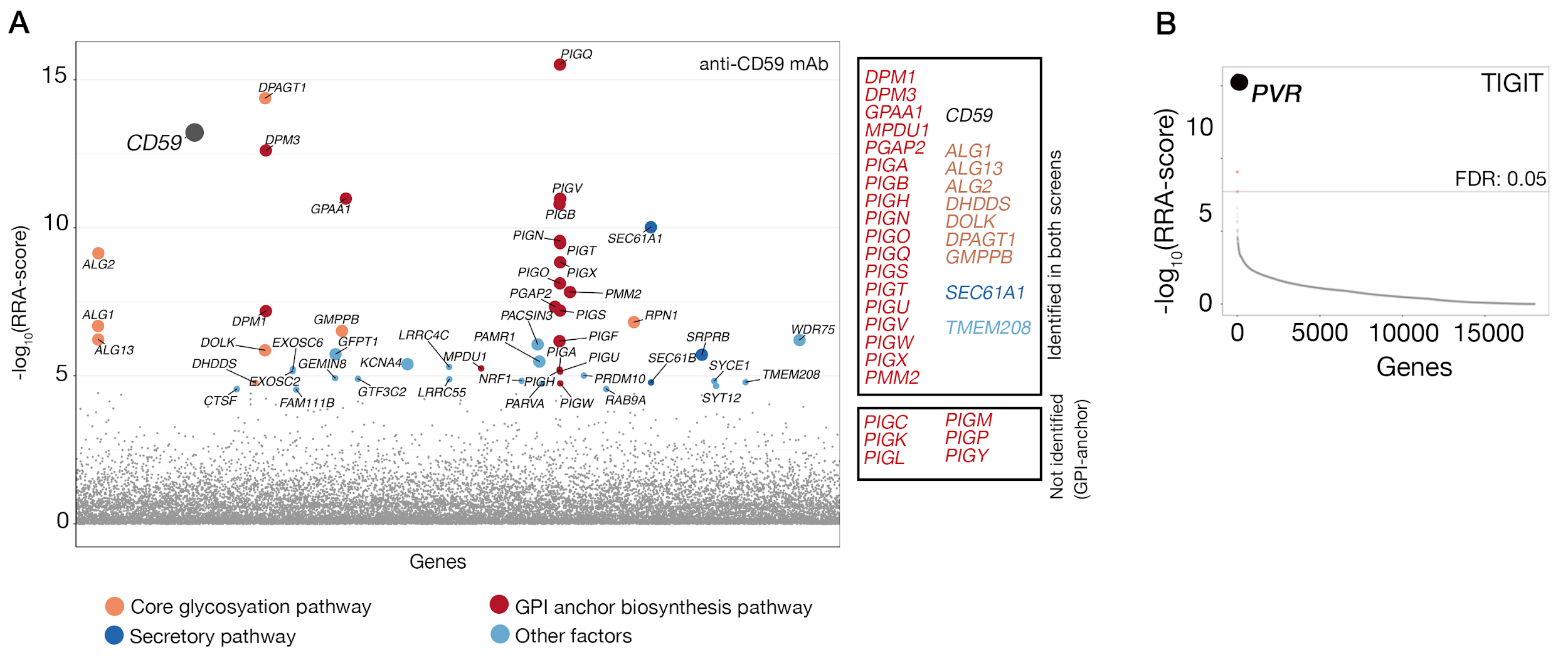


**Supplemental Figure S3: The experimental parameters established to identify cell surface receptor interactions using a cell-based CRISPR screening approach are robust and repeatable.** The genome-wide CRISPR screening approach was independently repeated to identify genetic pathways required for the cell surface display of CD59 using a high affinity monoclonal antibody. The cell surface display of CD59 involved many genes with a range of effect sizes in the GPI-anchor biosynthesis, N-linked glycosylation, and general secretory pathways. The majority of GPI-anchor biosynthesis genes detected in the initial screen were again detected with FDR < 0.05 in the repeat experiment (genes named in the upper boxed section) with only 6 out of 24 genes previously identified in this pathway (named in the lower boxed section) falling below this threshold.

**
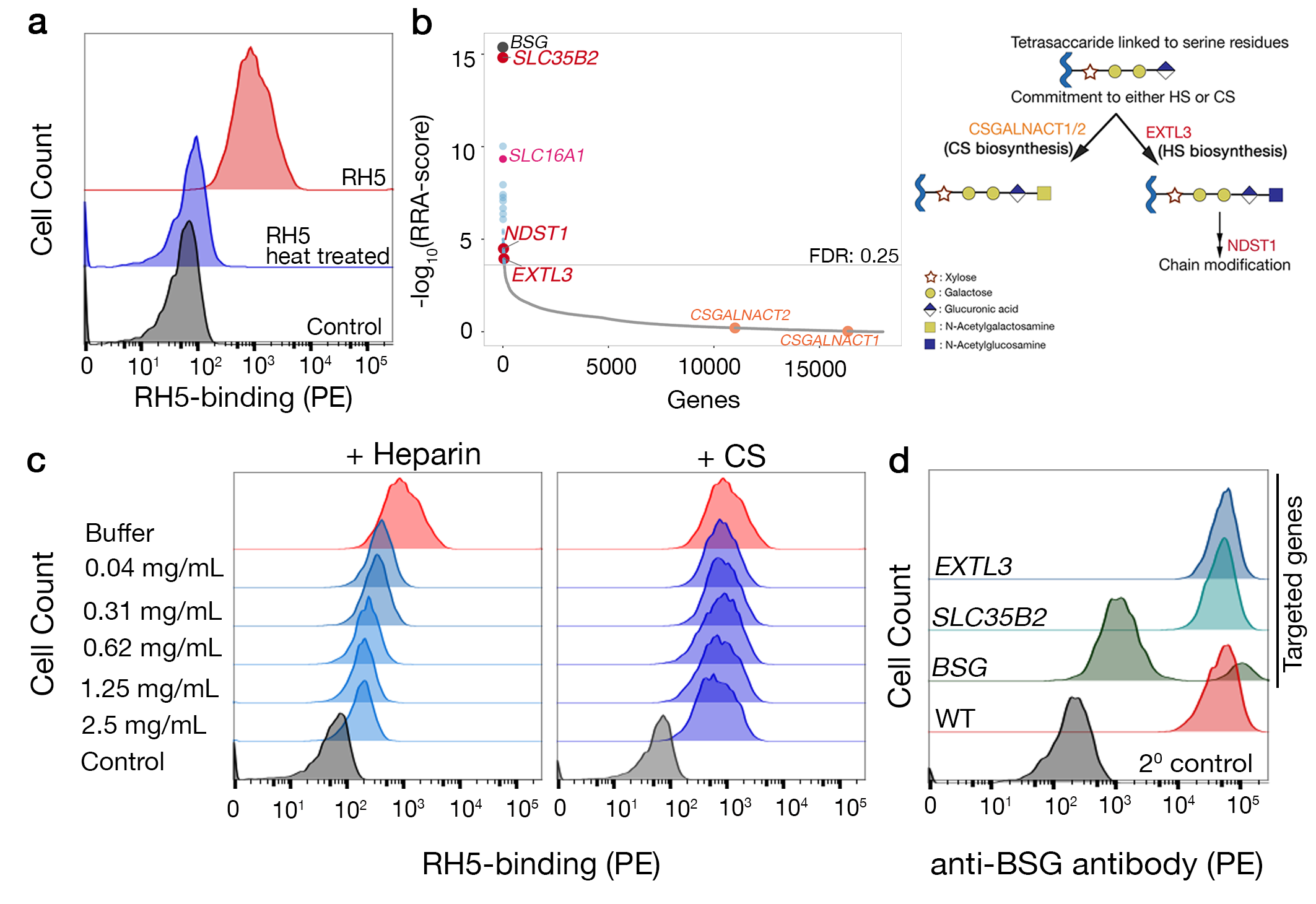
Supplemental Figure S4: RH5 binding to HEK293 cells was heat labile and was partially blocked by preincubation with heparin, but not chondroitin sulfate.** **a**. Biotinylated RH5 clustered around streptavidin-PE bound HEK293 cells. Heat treatment (80 ^o^C for 10 minutes) of biotinylated RH5 prior to streptavidin clustering abrogated all binding back to a negative control; a representative of three independent experiments is shown. **b**. Genes required for the biosynthesis of heparan sulfate and not chondriotin sulfate are required for RH5 binding. Analysis of gRNAs in sorted mutant cells that had lost binding to a fluorescently labelled RH5 probe (left panel) demonstrated that genes encoding enzymes that are critical for HS biosynthesis (*EXTL3* and *NDST1*) but not CS biosynthesis (*CSGALNACT1* and *CSGALNACT2*) were enriched. The general GAG biosynthesis pathway bifurcates into HS and CS biosynthesis pathway after the formation of a linkage tetrasaccharide structure (right panel). Consistent with this, a broader pathway analysis involving all the identified genes using KEGG-annotated pathways identified the HS but not CS biosynthesis pathway as significantly enriched (FDR = 0.001). **c**. Clustered RH5 binding probe was pre-incubated with the indicated concentrations of either heparin or chondroitin sulfate (CS) prior to presentation to HEK293 cells and binding quantified by flow cytometry. Preincubation of RH5 with heparin showed a dose-dependent inhibition of binding up to a threshold; preincubation with CS showed no inhibition of RH5 binding, even at the maximum concentration of 2.5 mg/mL. A representative of three independent experiments is shown. **d**. Cells transduced with lentivirus encoding gRNAs targeting enzymes in the heparan sulfate biosynthesis pathway show no alteration in surface BSG expression. Cell surface levels of BSG were quantified by flow cytometry on parental HEK293 cells or those transduced with lentivirus encoding single gRNAs targeting *SLC35B2* or *EXTL3* (genes required for HS biosynthesis), or *BSG*, as a control. Cells were stained with an anti-BSG mAb; control cells are stained with secondary antibody alone. A representative of two independent experiments is shown. In **a** and **c**, the control represents binding of biotinylated protein tags (Cd4d3+4 alone) clustered around streptavidin-PE to the parental HEK293 cell line.

**
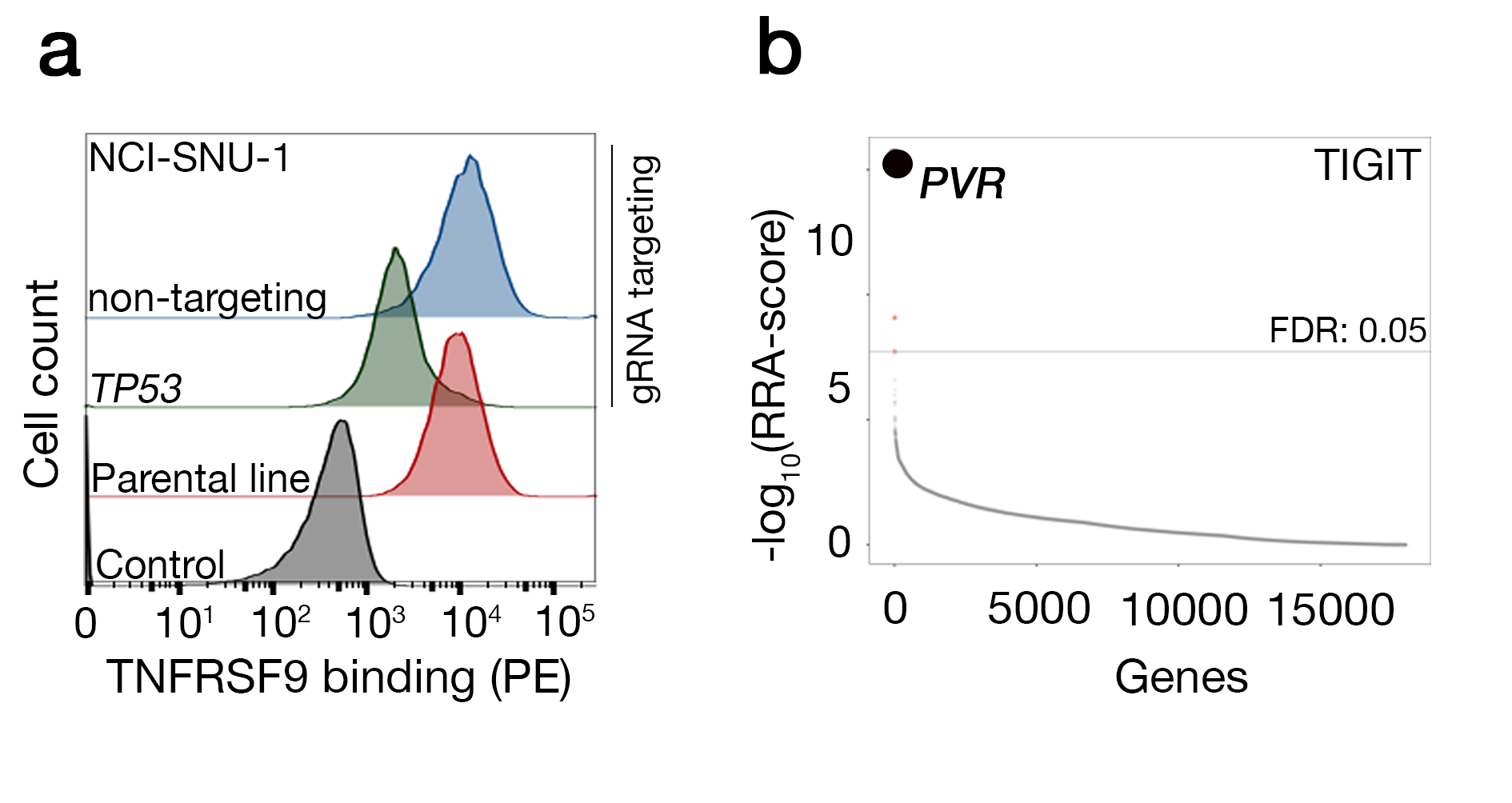
**

**Supplemental Figure S5: Validation that *TP53* contributes to binding of TNFRSF9 to NCI-SNU-1 cells and repeated screen with a low-affinity ligand demonstrating the robustness of the screening parameters**. **a.** TNFRSF9 binding to NCI-SNU-1 cells was reduced in *TP53*-targeted cells relative to a non-targeting control; targeted cells were maintained as polyclonal lines. Data shown is a representative experiment of three technical replicates. **b.** An independent replicate for a low affinity cell surface receptor-ligand interaction using the TIGIT probe again unambiguously identified its receptor, PVR, with very high statistical confidence.

**
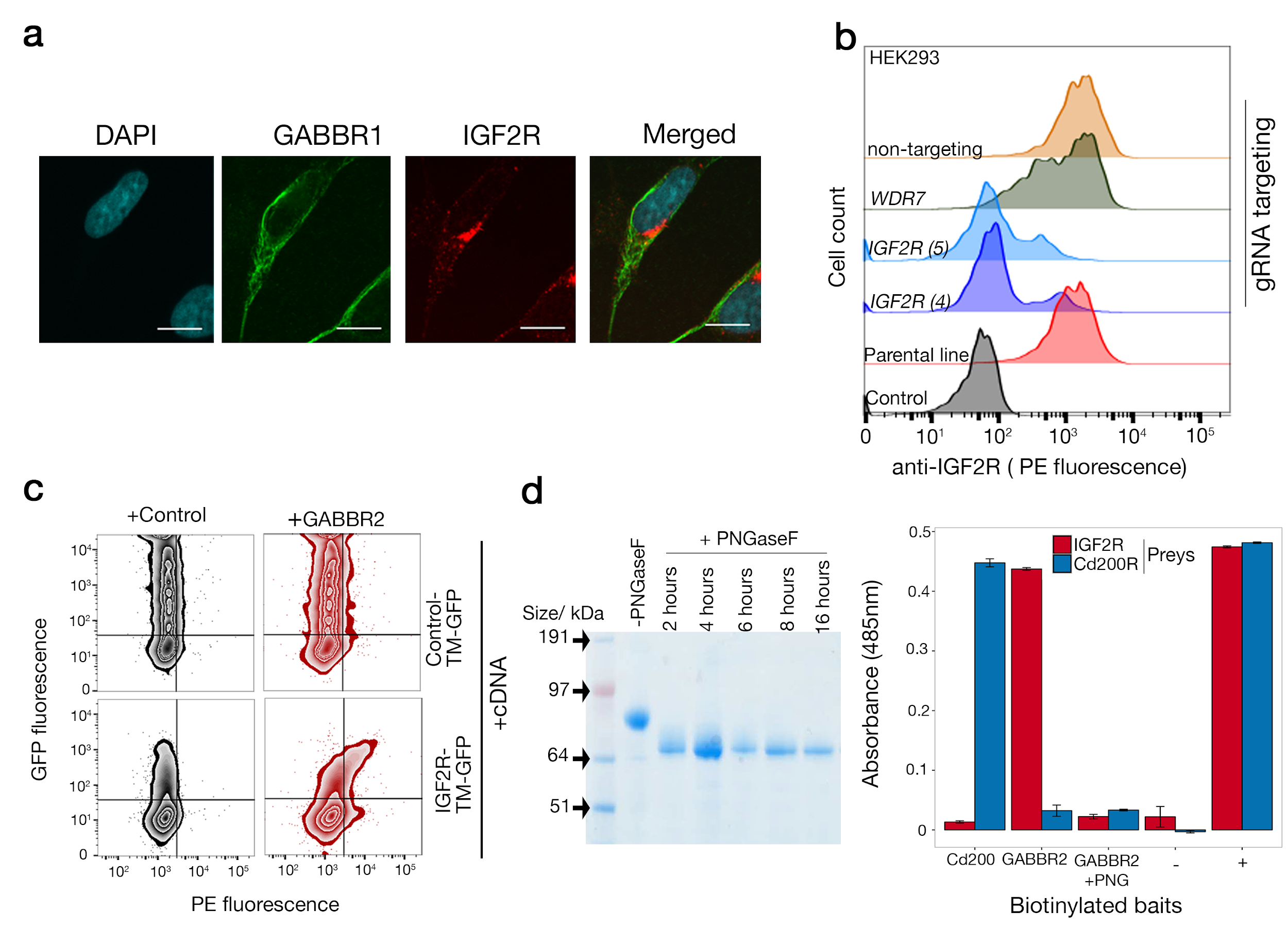
**

**Supplemental Figure S6: Membrane localization of IGF2R and GABA_B_ receptor complex, validation of gRNAs targeting *IGF2R* and *WDR7*, and biochemical analysis of the IGF2R-GABBR2 interaction. a.** The GABA_B_ receptor complex is localized both in the plasma membrane and also in the intracellular membranous compartments of the SK-N-SH neuronal cell line. IGF2R is mainly localized in the perinuclear vesicular compartments, but distinct punctate staining is also observed on the plasma membrane. The GABA_B_ receptor complex was localized with mouse anti-GABBR1 (secondary antibody: goat-anti mouse Alexa 488, in green) and IGF2R with rabbit anti-IGF2R (secondary antibody: donkey anti-rabbit Alexa 647, in red). Nuclei are counterstained with DAPI (cyan). Scale bars represent 10 µm. Shown is a representative confocal microscopy image from three independent experiments. **b.** HEK293-Cas9 cells transduced with lentiviruses encoding individual gRNAs targeting *IGF2R* (two different gRNAs labeled (4) and (5) ) and *WDR7* show reductions in cell surface IGF2R levels. HEK293-Cas9 cells were transduced with lentiviruses encoding the indicated gRNAs and stained 12 days later with a mouse anti-human IGF2R mAb. Cells stained with secondary antibody alone were the negative control and WT indicates untransduced cells as a positive control. A representative of three technical replicates is shown. **c.** Gain of GABBR2 binding in cells transfected with a cDNA encoding IGF2R. NCI-SNU-1 cells (which do not display IGF2R on their surface) were transfected with either a cDNA construct encoding the entire ectodomain of IGF2R fused to transmembrane region and an intracellular eGFP or a control TM-GFP tagged receptor and tested for their ability to bind a fluorescently labelled GABBR2 binding probe; only the IGF2R-GFP positive cells and not the GFP negative or control-TM-GFP bound GABBR2. **d**. Treating GABBR2 with PNGaseF ablates the interaction with IGF2R. Left panel: The purified ectodomain of GABBR2-Cd4d3+4-bio was treated with PNGaseF for the indicated times at 37 ºC before aliquots were taken, resolved by SDS-PAGE under reducing conditions and stained with Coomassie Blue. No further reductions in molecular mass due to PNGaseF treatment occurred after eight hours incubation suggesting the vast majority of glycans had been removed from the protein. Right panel: The indicated biotinylated bait proteins were immobilised on a streptavidin-coated microtitre plate and probed for interactions with the beta-lactamase-tagged prey proteins. Prey binding was quantified by measuring the absorbance of the hydrolysis products of nitrocefin - a beta-lactamase substrate - at 485 nm. The known interaction between Cd200-Cd200R was used as the positive control in the assay. Negative control (-) was prey capture by a biotinylated Cd4d3+4 tag only control and positive control (+) was total capture of all preys with an anti-prey antibody. Bars represent means ± s.e.m., *n* = 3. In **a** and **b** data is representative of three technical replicates.


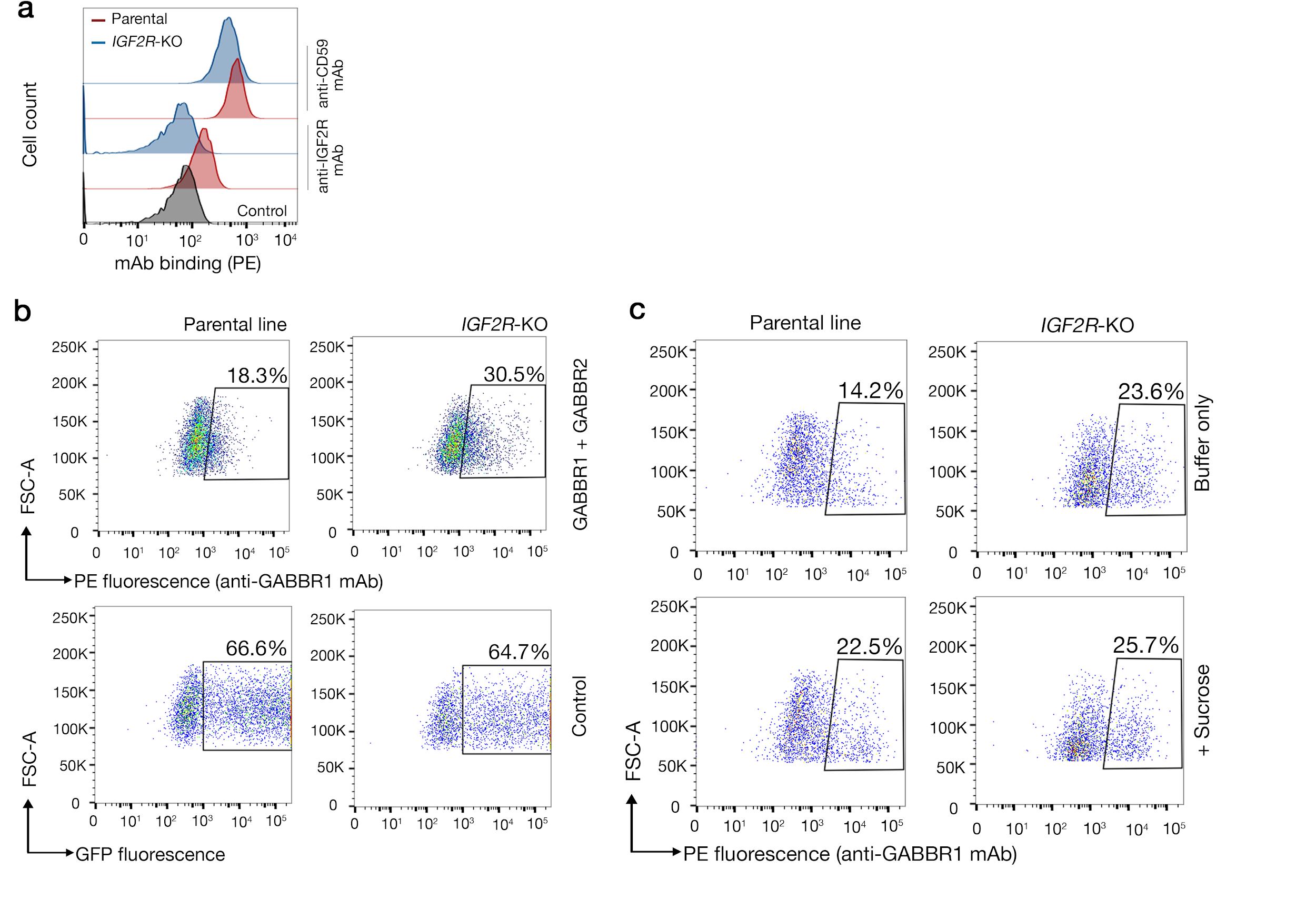


**Supplemental Figure S7: Cell-based functional assay in parental and *IGF2R*-KO HEK293 cells demonstrate that IGF2R internalizes GABA_B_ receptor complex in a clathrin dependent manner. a.** Clonal line generated by targeting *IGF2R* showed a complete absence of IGF2R from the surface of HEK293 cells. The cell surface expression level of an unrelated protein, CD59, was not significantly changed by knocking out IGF2R. **b.** Examples of the raw FACS data used to assess the functional interaction of the GABA_B_ receptor complex with IGF2R. Flow cytometry plots quantifying the level of GABA_B_ receptor complex on the surface of parental HEK293-Cas9 cells (top left) compared to the IGF2R-deficient cells (top right); the gated regions are shown and were selected using a negative staining control. Transfection of parental HEK293-Cas9 cells (bottom left) and IGF2R-deficient cells (bottom right) with a control receptor fused to GFP shows that both cell lines were transfected with equal efficiency. **c.** Examples of raw FACS data analysed in Figure 5c. Flow cytometry plots quantifying the level of GABA_B_ receptor complex on the surface parental HEK293-Cas9 cells (top left) compared to the IGF2R-deficient cells (top right); the gated regions are shown and were selected using a negative staining control. Bottom panel depicts the surface level expression of GABA_B_ receptor complex in parental (left) and *IGF2R*-KO (right) cells upon treatment with sucrose.

**
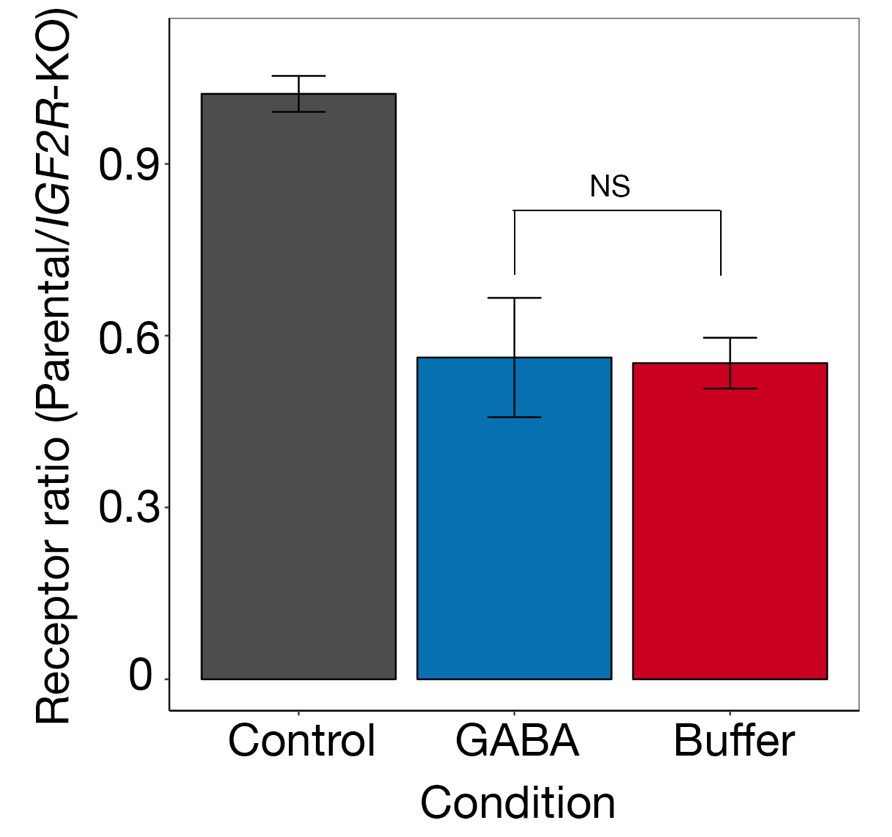
**

**Supplemental Figure S8: Addition of the agonist, GABA, has no effect on the interaction between IGF2R and the GABAB receptor complex.** Parental and *IGF2R*-deficient HEK293 cells expressing the GABAB receptor complex were stimulated for an hour with GABA (100μM) before measuring the cell surface receptor levels using an anti-GABBR1 monoclonal antibody and flow cytometry. The effect of GABA on the IGF2R-GABAB-receptor complex interaction was determined by calculating the ratio of positively stained cells on the parental cell line relative to the IGF2R-deficient cells, relative to a buffer only control. Control represents transfection control. Bars represent mean ± s.d.; *n* = 3.

**
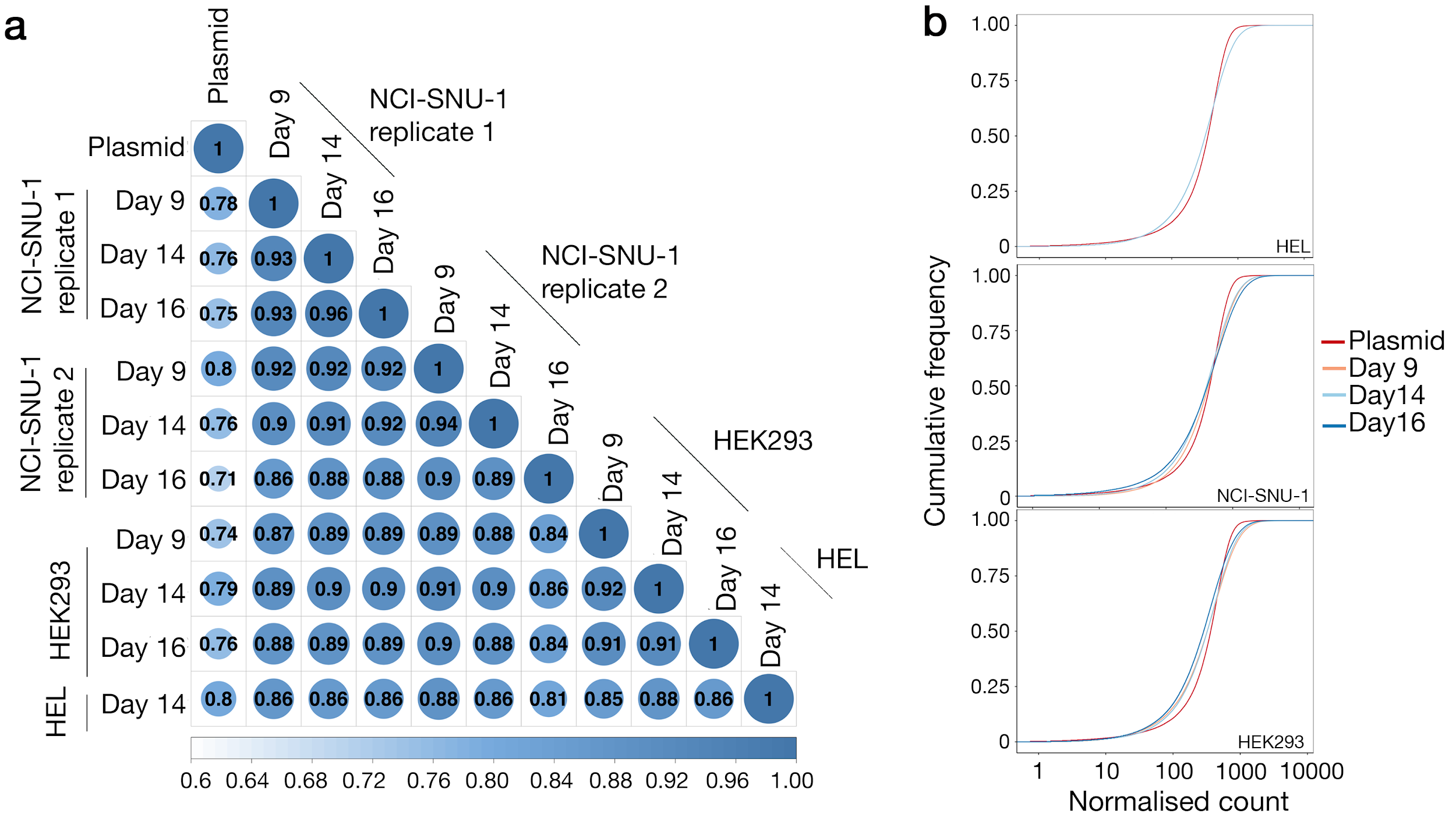
**

**Supplemental Figure S9: Genome-scale libraries of mutant cells are reproducibly created using CRISPR-Cas9 technology and lentiviral gRNA delivery**. **a**. Matrix plot depicting the correlation between normalized read counts of all gRNAs in the original plasmid library and those amplified from gene knockout libraries from different cell lines and days. The correlation between the two biological replicates of NCI-SNU-1, and between any cell line on any day was higher than when compared to the original plasmid pool. **b**. Cumulative distribution function plots comparing the gRNA abundance between the plasmid library to the mutant libraries of HEK293 and NCI-SNU-1 cells on days 9, 14 and 16 days, and HEL cells on day 14 post transduction. The differences in the curves from the mutant cell populations represent the depletion in a subset of gRNAs compared to the plasmid library.

**
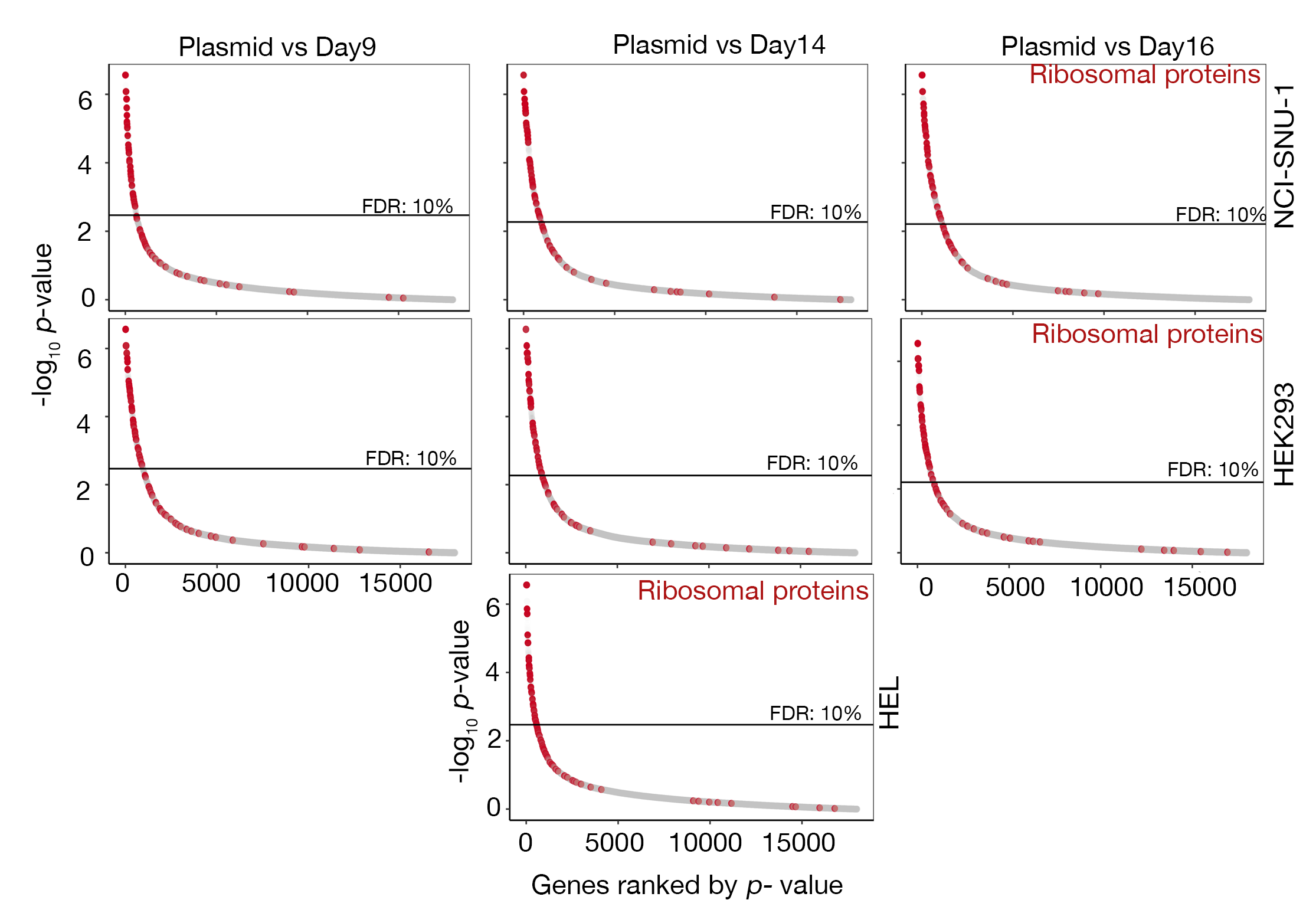
**

**Supplemental Figure S10: gRNAs targeting genes involved in essential biological processes were depleted during culture of the mutant cell library.** Gene-level enrichment analysis revealed genes involved in ribosomal biosynthesis among the most significantly depleted genes in NCI-SNU-1, HEL and HEK293 cell lines on all three time-points, days 9,14 and 16. For NCI-SNU-1, representative analysis from one of the two experiments is shown.

**Supplemental Table S1: False discovery rate (FDR) for identification of the genes encoding the known antibody target in a genome-scale screening approach.** Seven screens were carried out in this study using mAbs targeting five structurally diverse cell surface receptors. These included type I (GYPA, CD58), GPI anchored (CD59), a heterodimeric (integrin ɑ2β1) and multipass membrane (CD47) receptors. In all cases, the FDR of identification of direct receptor was lower than 0.05.

| **mAb clone name** | **Target** | **Identified receptor gene (FDR)** | **Receptor structural architecture** | **Antibody**  **Isotype** |
| --- | --- | --- | --- | --- |
| BRIC229 | CD59 | *CD59* (0.0002) | GPI anchor | Mouse IgG2b |
| BRIC229 (repeat) | CD59 | *CD59 (*0.00035) | GPI anchor | Mouse IgG2b |
| BRIC126 | CD47 | *CD47* (0.004) | Multi-membrane spanning (5-TM) membrane receptor | Mouse IgG2b |
| B6H12 |  | *CD47* (0.0016) |  | Mouse IgG2a |
| BRIC5 | CD58 (LFA-3) | *CD58* (0.0012) | Two isoforms identified:  Isoform 1: Single-pass type I membrane protein.  Isoform 2: GPI-anchor. | Mouse IgG2a |
| BRIC256 | GYPA | *GYPA* (0.0001) | Erythroid cell specific protein with 15 O-glycosylation and 1 N-glycosylation | Mouse IgG1 |
| P16 | Integrin ɑ2β1 | *ITGB1* (0.024)  Components of the Arp2/3 complex:  *ARPC4* (0.0007)  *ACTR2* (0.001)  *ARPC3* (0.04) | Integrin heterodimer | Mouse IgG1 |

**Supplemental Table S2: Protein expression plasmids used in this study**

| **Protein** | **Reference** |
| --- | --- |
| Plasmids used for expression biotinylated “monomers” containing C-terminal rat Cd4d3+4, biotinylation peptide and 6xHis tag. | |
| CD200R | Addgene plasmid # 36153 |
| RH5 | Addgene plasmid #47780 cloned into Addgene plasmid #50824 backbone |
| TNFRSF9 | Gene synthesis (MGNS….QIIS) using Geneart and insert cloned into Addgene plasmid # 32404 backbone |
| CRTAM | Gene synthesis (MWWR….KKSG) using Geneart and insert cloned into Addgene plasmid # 32404 backbone |
| TIGIT | Gene synthesis (MRWC….FQIP) using Geneart and insert cloned into Addgene plasmid #  32404 backbone |
| Syn1-RBD | M1-183P amplified from cDNA construct (Origene-RC213951) with primers: 5’ GACTGCGGCCGCCACCATGGCCCTCCCTTATCATA 3’ and 5’ TGAGGTCTCGGCCCAAAACCCTGGCGCGCCACTG 3’ and cloned into Addgene plasmid # 36153 backbone |
| Plasmids used for expression of Flag-tagged “pentamers” containing C-terminal Cd4d3+4, beta-lactamase, 3xFLAG tag and 6xHis tag. | |
| CD200R | Addgene plasmid # 71471 |
| EPHB1 | Addgene plasmid # 51750, cloned into Addgene plasmid # 71471 backbone |
| CD226 | Addgene plasmid # 51646 , cloned into Addgene plasmid # 71471 backbone |
| Plasmids used GABBR2-IGF2R study | |
| CD200 | Addgene plasmid # 32404 |
| CD200R | Addgene plasmid # 32407 |
| GABBR2 | Gene synthesis (M1-P480) and cloned into Addgene plasmid #36153  backbone |
| IGF2R-Prey | M1-2306V amplified from cDNA construct (Origene-SC300143) with primers: 5’GACTGCGGCCGCCACCATGGGGGCCGCCGCCGGCCGGAG 3’ and 5’ CAGTGGCGCGCCGACTGCCTGGCTCCGTTCTGAC 3’ and cloned into Addgene plasmid # 3240 backbone |
| IGF2R-TM-GFP | PCR amplified fragment of IGF2R ectodomain cloned into backbone vector with -TM-GFP [^1^](https://paperpile.com/c/CtvidH/RAcfT) |
| GABBR2-full length cDNA | Origene SC316628 |
| GABBR1-full length cDNA | Origene SC112876 |

**Supplemental Table S3: Sequences of guide RNAs used in this study**

| **Gene** | **gRNA** | **Oligos: Sense (S) and antisense (AS)- 5’-3’** | **gRNA ID from Human v1 library** |
| --- | --- | --- | --- |
| *BSG* | GTCGTCAGAACACATCAACG | S: CACCGTCGTCAGAACACATCAACG | BSG_CCDS12032.1_ex2_19:580670-580693:+_9-4 |
|  |  | AS: AAACCGTTGATGTGTTCTGACGAC |  |
| *EXTL3* | GGTCCATCCGTCCGCCAGTG | S: CACCGGTCCATCCGTCCGCCAGTG | EXTL3_CCDS6070.1_ex0_8:28574365-28574388:-_5-3 |
|  |  | AS: AAACCACTGGCGGACGGATGGACC |  |
| *SLC35B2* | GTCGGCGCAGCTACGAACAC | S: CACCGTCGGCGCAGCTACGAACAC | SLC35B2_CCDS34462.1_ex0_6:44223027-44223050:-_5-1 |
|  |  | AS: AAACGTGTTCGTAGCTGCGCCGAC |  |
| *TP53* | GCAGTCACAGCACATGACGG | S: CACCGCAGTCACAGCACATGACGG | TP53_CCDS11118.1_ex6_17:7578415-7578438:-_5-1 |
|  |  | AS: AAACCCGTCATGTGCTGTGACTGC |  |
| *IGF2R(4)* | GCGGTCACTACGCATTCCAG | S: CACCGCGGTCACTACGCATTCCAG | IGF2R_CCDS5273.1_ex16_6:160468883-160468906:-_5-4 |
|  |  | AS: AAACCTGGAATGCGTAGTGACCGC |  |
| *IGF2R(5)* | GACAACGACGGATACAGACC | S: CACCGACAACGACGGATACAGACC | IGF2R_CCDS5273.1_ex19_6:160477535-160477558:+_5-5 |
|  |  | AS: AAACGGTCTGTATCCGTCGTTGTC |  |
| *WDR7* | GTGCGGAATGAATCACTAGC | S: CACCGTGCGGAATGAATCACTAGC | WDR7_CCDS11962.1_ex7_18:54358952-54358975:-_5-5 |
|  |  | AS: AAACGCTAGTGATTCATTCCGCAC |  |

**Supplemental Table S4: Summary of the screening parameters for all screens carried out in this study.** The table indicates the total number of cells screened within each library, the day the selection was performed, the number of collected cells, and the sorting threshold each for each indicated staining probe/cell line pairing used.

| **Cell line** | **Staining probe** | **Day of selection** | **Number of sorted cells in the library (x10^6^)** | **Number of collected cells** | **Sorting threshold (% of non-binding cells selected)** |
| --- | --- | --- | --- | --- | --- |
| HEK293 | BRIC229 | 15 | 100 | 380,000 | 0.5 |
|  | BRIC229 (repeated) | 15 | 100 | 340,000 | 0.45 |
|  | BRIC126 | 9 | 80 | 280,000 | 1 |
|  | B6H12 | 9 | 80 | 200,000 | 1 |
|  | MEM6/6 | 16 | 60 | 40,000 | 0.2 |
|  | BRIC5 | 9 | 50 | 120,000 | 0.8 |
|  | P16 | 9 | 60 | 180,000 | 1 |
|  | CD226 | 9 | 70 | 340,000 | 1.7 |
|  | EPHB1 | 9 | 70 | 300,000 | 1.4 |
|  | CRTAM | 14 | 70 | 500,000 | 1 |
|  | RH5 | 9 | 80 | 200,000 | 0.7 |
|  | TIGIT | 14 | 110 | 750,000 | 1.2 |
|  | TIGIT (repeated) | 16 | 100 | 700,000 | 1.1 |
|  | Syn1 | 9 | 100 | 800,000 | 1.5 |
|  | GABBR2 | 14 | 125 | 780,000 | 2.3 |
| HEL | BRIC256 | 14 | 50 | 300,000 | 1.7 |
| NCI-SNU-1 | TNFRSF9 | 14 | 80 | 600,000 | 1 |

**Supplemental Table S5. Summary of the relative gRNA abundances targeting the identified receptor in the different screens within this study.** The receptor gene identified using the indicated protein probe is indicated together with the ranked abundance position of sequencing reads for each of the gRNAs targeting that gene.

| Monoclonal antibodies | | | |
| --- | --- | --- | --- |
| Antibody / protein probe | Identified Receptor | Read count abundance ranking (out of 90,709 total) for each of the five gRNAs targeting the identified receptor | Number of sgRNA with rank below the alpha cutoff.^*^ |
| Anti-CD58 | *CD58* | 1, 16, 18, 32, 3184 | 5/5 |
| Anti-CD59 | *CD59* | 1, 12, 13, 45, 76 | 5/5 |
| Anti-CD59 (repeated) | *CD59* | 2, 8, 41, 55, 315 | 5/5 |
| Anti-GYPA | *GYPA* | 2, 24, 100, 34370 | 3/4 |
| Anti-CD47 (BRIC126) | *CD47* | 1, 8, 9182, 55700, 55701 | 3/5 |
| Anti-CD47 (B6H12) | *CD47* | 1, 20, 30047, 32120, 73483 | 4/5 |
| Anti-ITGB1 | *ITGB1* | 3, 34, 415, 4246, 36465 | 5/5 |
| Anti-BSG | *BSG* | 1, 2, 3, 5, 14, 38, 42, 816, 6308** | 9/9 |
| Recombinant protein probes | | | |
| PfRH5 | *BSG* | 1, 3, 19, 32, 39, 107, 914, 6199, 9586** | 9/9 |
| CRTAM | *CADM1* | 1, 2, 3, 4, 6 | 5/5 |
| TIGIT | *PVR* | 1, 2, 3, 5, 14 | 5/5 |
| TIGIT-repeated | *PVR* | 1, 2, 3, 4, 34 | 5/5 |
| CD226 | *PVR* | 1, 2, 4, 6, 9 | 5/5 |
| TNFSFR9 | *TNFSF9* | 5, 7, 8, 9, 14 | 5/5 |
| EPHB1 | *EFNB2* | 7, 62, 124, 174, 2269 | 5/5 |
| Syn1-RBD | *SLC5A1* | 1, 2, 16, 142, 365 | 5/5 |
| GABBR2 | *IGF2R* | 2, 5, 11, 20, 35 | 5/5 |

*The alpha cutoff in MAGeCK is determined by the FDR threshold for gene test, which using default parameter of MAGeCK is 0.25.

** There are nine gRNAs targeting *BSG*
